# Characterization, expression patterns and functional analysis of the MAPK and MAPKK genes in watermelon (*Citrullus lanatus*)

**DOI:** 10.1186/s12870-015-0681-4

**Published:** 2015-12-23

**Authors:** Qiuming Song, Dayong Li, Yi Dai, Shixia Liu, Lei Huang, Yongbo Hong, Huijuan Zhang, Fengming Song

**Affiliations:** 1 State Key Laboratory for Rice Biology, Institute of Biotechnology, Zhejiang University, Hangzhou, 310058 P. R. China; 2 College of Life Science, Taizhou University, Taizhou, Zhejiang 318001 P. R. China

**Keywords:** Watermelon (*Citrullus lanatus*), Mitogen-activated protein kinase cascade, ClMPK, ClMKK, Protein-protein interaction, Expression patterns, Transient expression, Disease resistance

## Abstract

**Background:**

Mitogen-activated protein kinase (MAPK) cascades, which consist of three functionally associated protein kinases, namely MEKKs, MKKs and MPKs, are universal signaling modules in all eukaryotes and have been shown to play critical roles in many physiological and biochemical processes in plants. However, little or nothing is known about the MPK and MKK families in watermelon.

**Results:**

In the present study, we performed a systematic characterization of the ClMPK and ClMKK families including the identification and nomenclature, chromosomal localization, phylogenetic relationships, ClMPK-ClMKK interactions, expression patterns in different tissues and in response to abiotic and biotic stress and transient expression-based functional analysis for their roles in disease resistance. Genome-wide survey identified fifteen *ClMPK* and six *ClMKK* genes in watermelon genome and phylogenetic analysis revealed that both of the ClMPK and ClMKK families can be classified into four distinct groups. Yeast two-hybrid assays demonstrated significant interactions between members of the ClMPK and ClMKK families, defining putative ClMKK2-1/ClMKK6-ClMPK4-1/ClMPK4-2/ClMPK13 and ClMKK5-ClMPK6 cascades. Most of the members in the ClMPK and ClMKK families showed differential expression patterns in different tissues and in response to abiotic (e.g. drought, salt, cold and heat treatments) and biotic (e.g. infection of *Fusarium oxysporum* f. sp. *niveum*) stresses. Transient expression of *ClMPK1*, *ClMPK4-2* and *ClMPK7* in *Nicotiana benthamiana* resulted in enhanced resistance to *Botrytis cinerea* and upregulated expression of defense genes while transient expression of *ClMPK6* and *ClMKK2-2* led to increased susceptibility to *B. cinerea*. Furthermore, transient expression of *ClMPK7* also led to hypersensitive response (HR)-like cell death and significant accumulation of H_2_O_2_ in *N. benthamiana*.

**Conclusion:**

We identified fifteen *ClMPK* and six *ClMKK* genes from watermelon and analyzed their phylogenetic relationships, expression patterns and protein-protein interactions and functions in disease resistance. Our results demonstrate that ClMPK1, ClMPK4-2 and ClMPK7 positively but ClMPK6 and ClMKK2-2 negatively regulate the resistance to *B. cinerea* when transiently expressed in *N. benthamiana* and that ClMPK7 functions as a regulator of HR-like cell death through modulating the generation of H_2_O_2_.

**Electronic supplementary material:**

The online version of this article (doi:10.1186/s12870-015-0681-4) contains supplementary material, which is available to authorized users.

## Background

Mitogen-activated protein kinase (MAPK) cascades, which are widely distributed in eukaryotes, are highly conserved signaling modules downstream of receptors/sensors that transduce extracellular stimuli into intracellular responses [1, 2]. The MAPK cascades are composed of three sequentially acting protein kinases, namely MAPKK kinases (MEKKs), MAPK kinases (MKKs) and MAPKs (MPKs), and activated through the way of phosphorylation [1, 3]. In general, upon perception of the extracellular environmental and intracellular growth/developmental signals, the top kinases of the cascades, MEKKs, activate via phosphorylation their downstream MKKs, which in turn further phosphorylate MPKs [4]. In specific, the MKKs in the MAPK cascades act as dual-specificity kinases to activate MPKs through double phosphorylation of the T-x-Y motif in the activation loop. During this phosphorylation relay, the input signal can be amplified through the MAPK cascade and eventually the activated MAPKs modify via phosphorylation a set of specific downstream target proteins such as transcription factors and other signaling components leading to the activation of the expression of downstream genes [1, 4, 5].

During the last two decades, extensive genetic and biochemical studies have been performed to explore the functions of MAPK cascades in model plant species as well as in some economically important crops such as rice. These studies have demonstrated that the MAPK cascades and their individual components play critical roles in regulating growth/development and stress responses in plants. Furthermore, several functional intact MAPK cascades that are involved in growth/development and stress responses have been characterized biochemically [1, 2, 4]. For example, tobacco NPK1–NQK1–NRK1 and Arabidopsis YODA–MKK4/MKK5–MPK3/MPK6 play essential roles in cell division, whereas Arabidopsis MEKK1–MKK4/MKK5 –MPK3/MPK6 and MEKK1–MKK1/2–MPK4 act as positive or negative regulators of signaling pathways modulating the immune responses [1, 2, 6, 7].

The components of the MAPK cascades are generally composed of different gene families, namely MPK, MKK and MEKK families, which have been characterized at the genome-wide level in many plant species including Arabidopsis [8, 9], rice [9, 10], poplar [9], soybean [11], maize [12, 13], tomato [14–16], canola [17], banana [18], apple [19], *Gossypium raimondii* [20], mulberry [21] and *Brachypodium distachyon* [22]. The numbers of MPK and MKK families vary greatly across species. For example, there are 20 MPKs in Arabidopsis [8, 9], 17 in rice [9, 10], 19 in maize [13], 21 in poplar [9], 16 in tomato [14], 12 in canola [17], 10 in mulberry [21], 12 in grapevine [23], 17 in tobacco [24], 38 in soybean [11], 28 in *G. raimondii* [20] and 16 in *B. distachyon* [22]. Similarly, 10 MKKs in Arabidopsis [8, 9], 8 in rice [9], 9 in maize [12], 5 in tomato [15, 16] and in canola [17], 11 in soybean [11], 11 in poplar [9], and 12 in *B. distachyon* [22] were identified. Structurally, the MPKs contain eleven domains (I–XI) and the well conserved threonine and tyrosine residues existing between domains VII and VIII form the activation loop, which is thought to be phosphorylated for the activation of the MPKs [25]. It is well known that plant MPKs have two different activation loop motifs, either TEY or TDY; however, other novel activation loop variants were recently characterized in plants MPKs [26]. Generally, the MPKs can be divided into four groups based on phylogeny and the conserved TEY/TDY motifs and each group has been assigned different functions [8, 27]. Similarly, the MKKs can also be classified into four groups according to the S/T-x5-S/T domain and “D site” [8].

Watermelon (*Citrullus lanatus*) is one of important horticultural crops, providing favorite fresh fruits worldwide. However, little or nothing is known about the MPK and MKK families in watermelon so far. The recently completion of genome sequencing of watermelon [28] provides a powerful platform that makes it possible to characterize gene families at the genome-wide level. In the present study, we performed a genome-wide identification of the watermelon MPK and MKK families and carried out an extensive characterization of the ClMPK and ClMKK families in terms of the nomenclature, chromosomal distribution, the conserved motifs and phylogenetical relationships. We explored some selected members of the ClMPK and ClMKK families for their putative protein-protein interaction relationships, expression patterns among different tissues and in response to abiotic and biotic stresses and possible functions in disease resistance through transient expression-based functional analysis in *Nicotiana benthamiana*. Our characterization of the watermelon ClMPK and ClMKK families provides a useful platform for further functional studies of ClMPKs and ClMKKs in watermelon.

## Results

### Characterization of the ClMPK and ClMKK families in watermelon

To identify putative MPK and MKK genes in watermelon, we performed BLAST searches against the watermelon genome database using the well-characterized Arabidopsis AtMPKs and AtMKKs as queries and identified 15 and 6 non-redundant sequences that are putative MPK and MKK genes, respectively. The predicted amino acid sequences of the putative ClMPKs and ClMKKs were further examined by ExPASy Proteomics Server for the presence of the characteristic conserved domains. Overall, our systematic analyses revealed that the ClMPK and ClMKK families comprise of 15 and 6 members in the watermelon genome, respectively. For convenience, we assigned unique identities to each of the identified ClMPK and ClMKK genes with a two-letter code corresponding to *C. lanatus* (Cl), followed by the family name (MPK or MKK) and a number (Table 1) according to the Arabidopsis MPK and MKK nomenclature system [8]. Notably, the predicted loci Cla022002 (402 bp) and Cla022003 (867 bp), which are exactly the same to the loci CL08G09900 and CL08G09910 in PLAZA dicots 3.0 database (http://bioinformatics.psb.ugent.be/plaza/), were indeed the same gene encoding for ClMPK6 and encode polypeptides corresponding for 1–121 aa and 122–395 aa of AtMPK6. The coding sequence of *ClMPK6* was further confirmed by our cloning of the full-length cDNA using primers designed according to the predicted cDNA sequences of Cla022002 and Cla022003.

**Table 1:** Information on ClMPKs and ClMKKs in watermelon

Family	Genes	Loci	ORF (bp)	Size (aa)	MW (kD)	*p*I	T-loop	Group	EST no.	Full cDNA
MPK	ClMPK1	Cla022470	1161	386	44.67	6.35	TEY	C	1	Yes
	ClMPK3	Cla008291	1899	632	71.58	5.41	TEY	A	3	Yes
	ClMPK4-1	Cla011419	1152	383	44.01	6.47	TEY	B	3	Yes
	ClMPK4-2	Cla006629	1140	379	43.74	6.13	TEY	B	1	Yes
	ClMPK6	Cla022002+ Cla022003	1266	421	47.99	5.63	TEY	A	3	Yes
	ClMPK7	Cla014573	1107	368	42.57	6.67	TEY	C	2	Yes
	ClMPK9-1	Cla018932	1926	641	72.87	6.81	TDY	D	4	–
	ClMPK9-2	Cla004511	1422	473	54.42	6.80	TDY	D	2	Yes
	ClMPK9-3	Cla003498	1422	473	54.50	7.27	TDY	D	1	–
	ClMPK9-4	Cla018463	1554	517	59.19	8.44	TDY	D	1	–
	ClMPK13	Cla008298	1113	370	42.61	4.97	TEY	B	–	Yes
	ClMPK16	Cla009366	1686	561	63.85	8.66	TDY	D	1	Yes
	ClMPK19	Cla005389	1413	470	54.31	9.37	TDY	D	1	Yes
	ClMPK20-1	Cla005523	1893	630	70.70	9.01	TDY	D	4	–
	ClMPK20-2	Cla013487	1848	615	69.76	9.21	TDY	D	2	–
MKK	ClMKK2-1	Cla016842	1069	355	39.54	5.40	–	A	–	Yes
	ClMKK2-2	Cla011187	1023	340	38.13	5.26	–	A	5	Yes
	ClMKK3	Cla017119	1557	518	57.77	5.53	–	B	–	–
	ClMKK5	Cla012564	1110	369	41.45	8.91	–	C	2	Yes
	ClMKK6	Cla016802	1065	354	39.73	6.27	–	A	–	Yes
	ClMKK9	Cla018437	636	211	23.60	6.23	–	D	–	–

To assess whether the characterized *ClMPK* and *ClMKK* genes had expression support, we searched using the predicted cDNA sequences as queries against watermelon EST database (http://www.icugi.org/cgi-bin/ICuGI/tool/blast.cgi). The search results indicated that 14 ClMPK and 2 ClMKK genes had available EST supports (Table 1), representing 93.3 and 33.3 % of the *ClMPK* and *ClMKK* genes, respectively. We attempted to clone the full-length cDNAs of all *ClMPKs* and *ClMKKs* for the confirmation of the predicted sequences and for the functional and protein-protein interaction studies. However, we failed to amplify the full-length cDNAs for *ClMPK9-1*, *ClMPK9-3*, *ClMPK9-4*, *ClMPK20-1* and *ClMPK20-2*, which have EST supports, and for *ClMKK3* and *ClMKK9*, which do not have EST supports (Table 1). Ultimately, we amplified and cloned 10 *ClMPK* and 4 *ClMKK* genes, including *ClMPK13*, *ClMKK2-1* and *ClMKK6* that do not have EST supports (Table 1), for further studies in protein-protein interactions and functional analyses.

The sizes of the open reading frames (ORF) for the *ClMPK* genes range from 1107 bp (*ClMPK7*) to 1926 bp (*ClMPK9-1*) and accordingly the sizes of the encoded proteins range from 368 to 641 amino acids. The molecular weights of the ClMPK proteins are between 42.57 kD and 72.87 kD and the pIs range from 4.97 to 9.37 (Table 1). The predicted ClMKK9 is likely an incomplete MKK and lacks approximately 100 amino acids at the N-terminal when compared with its closest Arabidopsis homologue AtMKK9. The ORF sizes for the other five *ClMKK* genes range from 1023 bp (*ClMKK2-2*) to 1557 bp (*ClMKK3*) and accordingly the sizes of the encoded proteins range from 340 to 518 amino acids. The molecular weights of these ClMKK proteins are between 38.13 kD and 57.77 kD and the pIs range from 5.26 to 8.91 (Table 1).

### Structural features and phylogenetic analysis of the ClMPKs and ClMKKs

**Fig. 1:**
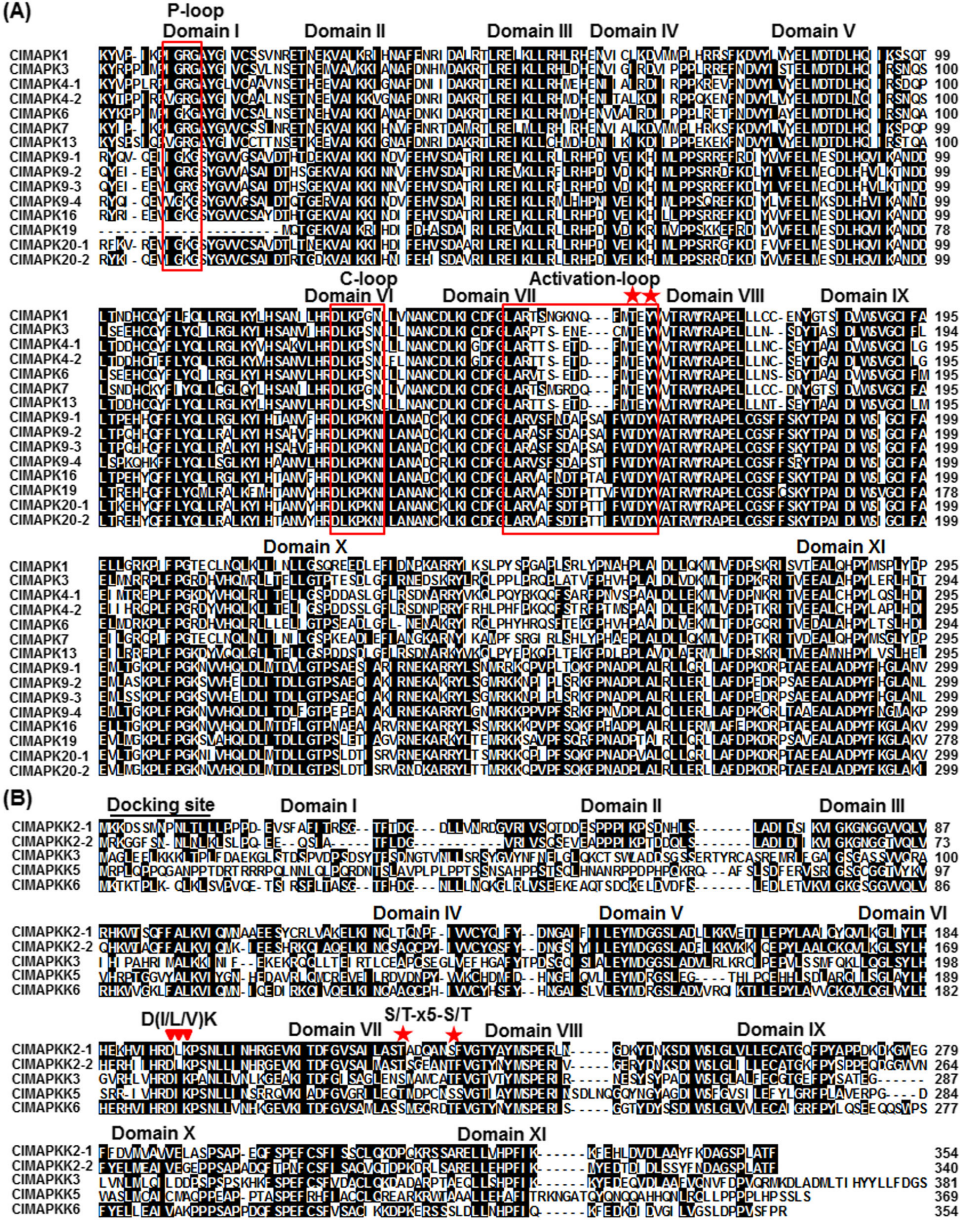
Sequence alignments and structural features of ClMPKs and ClMKKs. Multiple sequence alignment was performed using the ClustalX method and identical amino acids are shaded in black. The subdomains (I-XI) are indicated on the top of the aligned row. **a** Partial amino acid alignment of the 15 ClMPK proteins. The P-Loop, C-loop and activation-loop motifs are indicated with red boxes and the TxY motif is indicated by red stars. **b** Partial amino acid alignment of the 5 ClMKK proteins. The conserved S/T-x5-S/T motif and active site D(I/L/V)K motif are indicated by red stars and inverted red triangles, respectively. The docking site is indicated on the aligned row

**Fig. 2:**
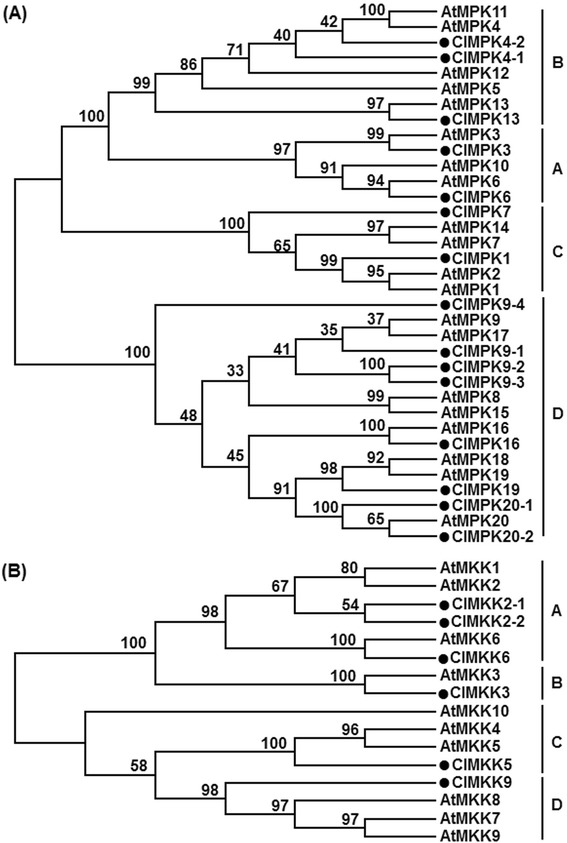
Phylogenetic analyses of ClMPKs and ClMKKs with Arabidopsis AtMPKs and AtMKKs. **a** Phylogenetic tree of ClMPKs. **b** Phylogenetic tree of ClMKKs. Phylogenetic trees were constructed by Neighbor-joining method using MEGA program and bootstrap values from 100 replicates are indicated at each node

Sequence alignment indicated that the ClMPK proteins contain highly conserved regions, spanning approximately 300 amino acids near the N-terminal portion, which are composed of eleven characteristic domains (I–XI) (Fig. 1a). Phylogenetic tree analysis with Arabidopsis AtMPKs revealed that the ClMPKs can be divided into four groups, namely A, B, C and D (Fig. 2a). Among 15 ClMPKs, ClMPK3 and ClMPK6 belong to Group A, ClMPK4-1, ClMPK4-2 and ClMPK13 are Group B members, only ClMPK1 falls into Group C, the other 8 members (ClMPK9-1, ClMPK9-2, ClMPK9-3, ClMPK9-4, ClMPK16, ClMPK19, ClMPK20-1 and ClMPK20-2) belong to Group D (Fig. 2a and Table 1). Several highly conserved characteristic motifs, e.g. activation-loop, P-loop and C-loop, were also identified in the ClMPK proteins (Fig. 1a). The activation-loop motifs are present between the domains VII and VIII and the TxY motif, which is phosphorylated for the activity, is present in all ClMPKs (Fig. 1a). Members in Groups A, B and C possess the TEY motif, whereas ClMPKs in Group D have the TDY motif (Fig. 1a and Table 1). However, no other TxY variant was found in all ClMPKs [14, 26]. In addition, a conserved CD domain with sequence of (LH)DxxDE(P)xC, which is thought to function as binding sites for upstream MKKs in the MAPK cascades [29], is present in Groups A and B ClMPKs but is absent in Group C and D ClMPKs. The TDY-containing ClMPKs have extended C-terminal regions, which are generally present in the TDY class of MPKs from other plants [8, 14, 18, 22]. In watermelon, there are 7 ClMPKs with TEY motif and 8 ClMPKs containing TDY motif (Table 1). This is similar to rice and *B. distachyon*, which contain more TDY-containing MPKs than the TEY-containing MPKs [9, 10, 22] but different from those in Arabidopsis, tomato, soybean and *G. raimondii*, which contain more TEY-containing MPKs than the TDY-containing MPKs [9, 11, 14, 20].

Sequence alignment revealed the ClMKKs except ClMKK9, which is an incomplete MKK, also contain 11 domains of protein kinases with serine/threonine specificity [9]. Conserved motifs were identified in ClMKKs. The characteristic S/T-x5-S/T motif between domains VII and VIII, which includes the serine/threonine residues whose phosphorylation is necessary for MKK activation, and active site D(I/L/V)K motif were conserved in ClMKKs (Fig. 1b). In addition, putative docking regions with characteristic sequence of K/R-K/R-K/R-x (1–6)-L-x-L/V/I were present in ClMKK2-1, ClMKK2-2, ClMKK3 and ClMKK6 (Fig. 1b). Phylogenetic tree analysis with Arabidopsis AtMKKs revealed that the ClMKKs can be divided into four groups, namely A, B, C and D (Fig. 2b). Among 6 ClMPKs, ClMKK2-1, ClMKK2-2 and ClMKK6 belong to Group A, whereas ClMKK3, ClMKK5 and ClMKK9 belong to Group B, C and D, respectively (Fig. 2b and Table 1). Similar to that in maize [12], the ortholog of AtMKK7/AtMKK8/AtMKK9 was not found in watermelon (Fig. 2b). Furthermore, the ClMKK family is relatively smaller than other plant species such as Arabidopsis (10 AtMKKs) [8], rice (8 OsMKKs) [9], maize (9 ZmMKKs) [12], soybean (11 GmMKKs) [11]; popular (13 PtMKKS) [9] and *B. distachyon* (12 BdMKKs) [22]. The relatively small ClMKK family in watermelon may be a consequence from species-specific diversification during evolution and implies that the ClMKK proteins may have evolved to possess pleiotropic effects in diverse biological processes.

### Genomic distribution and evolution of the *ClMPK* and *ClMKK* families

**Fig. 3:**
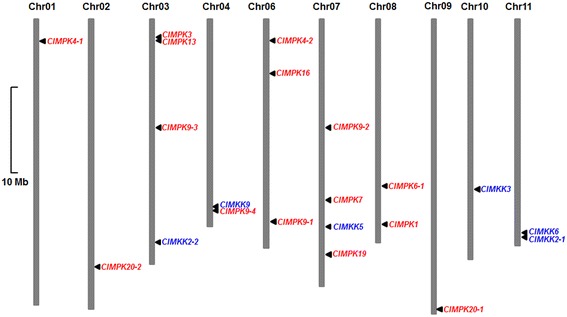
Chromosomal distribution of the *ClMPK* and *ClMKK* genes. The *ClMPK* and *ClMKK* genes are indicated in red and blue colors, respectively. Scale bar represents 10 Mb

The 15 *ClMPK* and 6 *ClMKK* genes were anchored on ten of the 11 watermelon chromosomes (Fig. 3). The chromosomal distribution pattern indicated that some chromosomes and chromosomal regions have a relatively high density of *ClMPK* or *ClMKK* genes, e.g. neither *ClMPK* nor *ClMKK* gene was located on chromosome 5. In the *ClMPK* family, one *ClMPK* gene is located on each of chromosomes 1, 2, 4 and 9; two *ClMPK* genes were found to be located on chromosome 8 and three *ClMPK* genes are distributed on each of the chromosomes 3, 6 and 7 (Fig. 3). In the *ClMKK* family,two *ClMKK* genes are located on chromosome 11 while only one *ClMKK* gene is located on each of the chromosomes 3, 4, 7 and 10 (Fig. 3). No gene cluster, as defined by the criteria that four or more genes are present within a region of 200 Kb or less on a chromosome [29], was found for the *ClMPK* and *ClMKK* families. However, five paralog pairs such as *ClMPK4-1*/*ClMPK4-2*, *ClMPK9-1*/*ClMPK9-4*, *ClMPK20-1*/*ClMPK20-2*, *ClMKK2-1*/*ClMKK2-2* and *ClMKK2-2/ClMKK5*, sharing high similarity in sequences, were distributed on different chromosomes (Fig. 3), indicating that they are not tandem duplicated gene pairs. Although *ClMPK3* and *ClMPK13* are tightly located on chromosome 3, they only share 65 % of identity at amino acid sequence level and are also not tandem duplicated genes. It is thus likely that tandem duplication plays a limited role in the evolution of the *ClMPK* and *ClMKK* genes. This is similar to the observations for the tomato *SlMAPK* and *SlMKK* families [14, 15].

### Interactions between ClMPKs and ClMKKs

**Fig. 4:**
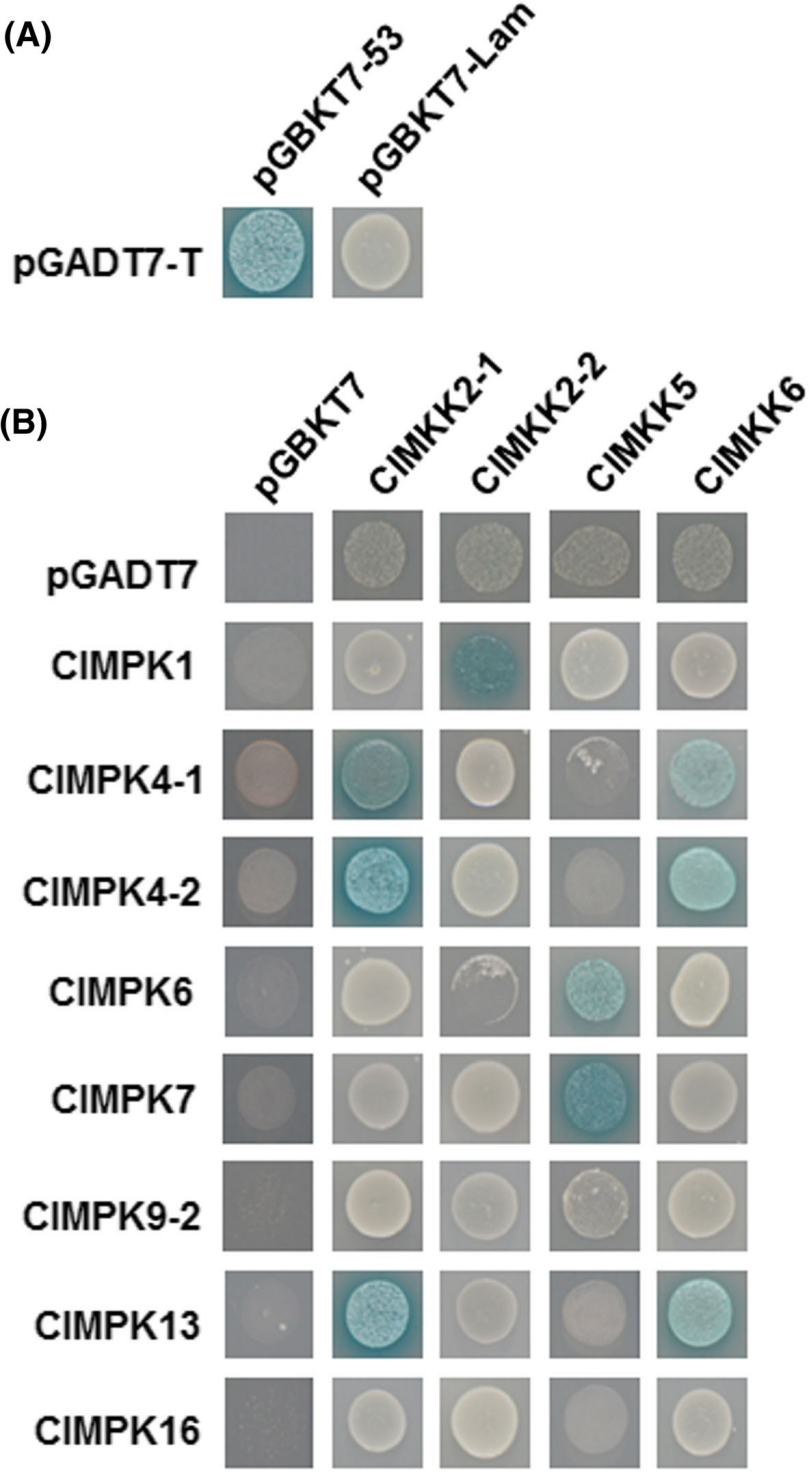
Interactions between selected ClMPKs and ClMKKs. **a** Positive (pGADT7-T + pGBKT7-53) and negative (pGADT7-T + pGBKT7-Lam) controls. **b** Interactions between selected ClMPKs and ClMKKs. Yeasts harboring the indicated plasmid combinations were grown on selective medium SD/Trp^−^His^−^ and β-galactosidase activity showing positive interactions was examined by addition of X-α-gal. Repeated experiments showed similar results

To examine the interactions and specificity between ClMPKs and ClMKKs, a series of yeast two-hybrid assays were performed to establish putative interaction relationships between ClMKKs and ClMPKs. For this purpose, four *ClMKK* genes (*ClMKK2-1*, *ClMKK2-2*, *ClMKK5* and *ClMKK6*) and eight *ClMPK* genes (*ClMPK1*, *ClMPK4-1*, *ClMPK4-2*, *ClMPK6*, *ClMPK7*, *ClMPK9-2*, *ClMPK13* and *ClMPK16*) were cloned into the respective DNA-binding domain and GAL4 activation domain plasmids, respectively. After co-transformation into the yeast strain YH2Gold, interactions were monitored by growth on selective medium and the production of blue pigment after addition of X-α-gal. In our experiments, a positive control (pGADT7-T + pGBKT7-53) and a negative control (pGADT7-T + pGBKT7-Lam) were always included to rule out possible false interaction (Fig. 4a). As shown in Fig. 4b, interactions between tested ClMPKs and ClMKKs were detected. ClMKK2-1 exhibited strong interactions with CllMAPK4-2, ClMPK13 and ClMPK4-1; whereas ClMKK2-2 had a significant interaction with ClMPK1 (Fig. 4b). Similarly, significant interactions between ClMKK6 and ClMPK4-1, ClMPK4-2 or ClMPK13 and between ClMKK5 and ClMPK6-1 or ClMPK7 were observed (Fig. 4b). Among the ClMPKs tested, ClMPK9-2 and ClMPK16 were not found to interact with any of the four ClMKKs, probably having interactions with other ClMKKs.

### Expression patterns of *ClMPK* and *ClMKK* genes

#### Tissue-specific expression patterns

**Fig. 5:**
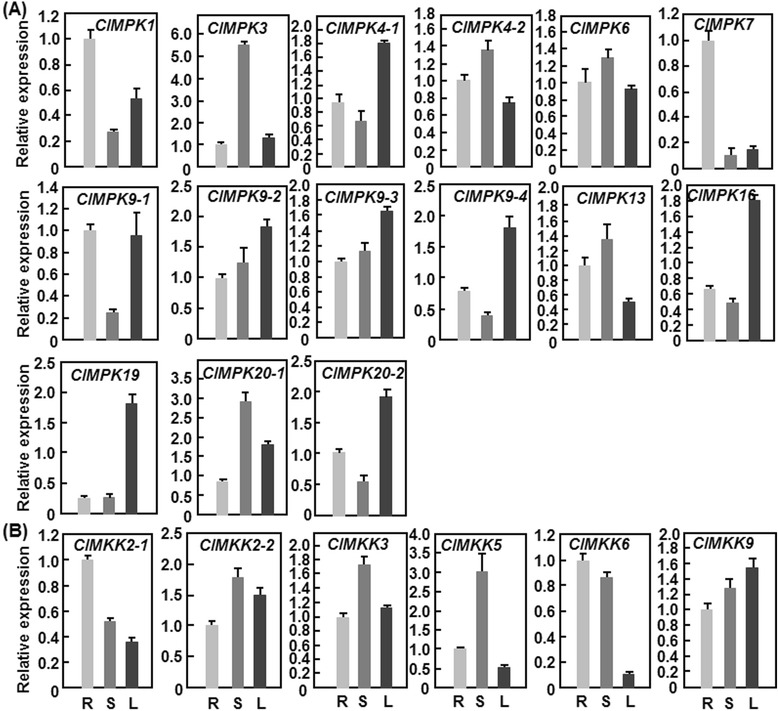
Expression patterns of *ClMPKs* (**a**) and *ClMKKs* (**b**) in roots, stems and leaves of watermelon plants. Root, stem and leaf samples were collected from 3-week-old plants and relative expression was shown as folds of the actin transcript values. Data presented are the means ± SD from three independent experiments

It is well known that MAPK cascades play critical roles in plants growth and development [2]. To gain insights into the involvement of the *ClMPK* and *ClMKK* genes in growth and development, we analyzed by quantitative reverse transcription PCR (qRT-PCR) their tissue-specific expression patterns in three different tissues such as roots, stems and leaves from 3-week-old watermelon plants. As shown in Fig. 5, the 15 *ClMPK* and 6 *ClMKK* genes were constitutively expressed in all tested tissues but exhibited different expression patterns. In the *ClMPK* family, *ClMPK9-1*, *ClMPK1* and *ClMPK7* in roots, *ClMPK20-1*, *ClMPK3*, *ClMPK13*, *ClMPK4-2* and *ClMPK6* in stems, and *ClMPK9-3*, *ClMPK19*, *ClMPK16*, *ClMPK4-1*, *ClMPK9-4*, *ClMPK9-2* and *ClMPK20-2* in leaves showed the highest expression levels, whereas in the *ClMKK* family, the highest expression levels of *ClMKK6* and *ClMKK2-1* in roots, *ClMKK2-2*, *ClMKK3* and *ClMKK5* in stems and *ClMKK9* in leaves were observed (Fig. 5). Comparison of the expression patterns identified some tissue-specifically expressed *ClMPK* and *ClMKK* genes, e.g., *ClMPK3* having high expression level in stems but very low levels in roots and leaves, *ClMPK7* with high expression level in roots but very low levels in stems and leaves, *ClMPK19* showing high expression level in leaves but very low levels in roots and stems (Fig. 5a) and *ClMKK5* having high expression level in stems but very low levels in roots and leaves (Fig. 5b), indicating that *ClMPK3*/*ClMKK5*, *ClMPK7* and *ClMPK19* may play specific roles in stems, roots and leaves, respectively. Furthermore, the paralog pairs *ClMPK4-1*/*ClMPK4-2*, *ClMPK9-1*/*ClMPK9-4*, *ClMPK20-1*/*ClMPK20-2* and *ClMKK2-1*/*ClMKK2-2*, sharing high similarity in sequences, exhibited distinct expression patterns in roots, stems and leaves (Fig. 5), indicating that the high levels of expression of these genes in specific tissues may be determined by their biological functions rather than the sequence similarity.

#### Expression patterns in response to abiotic stresses and ABA

**Fig. 6:**
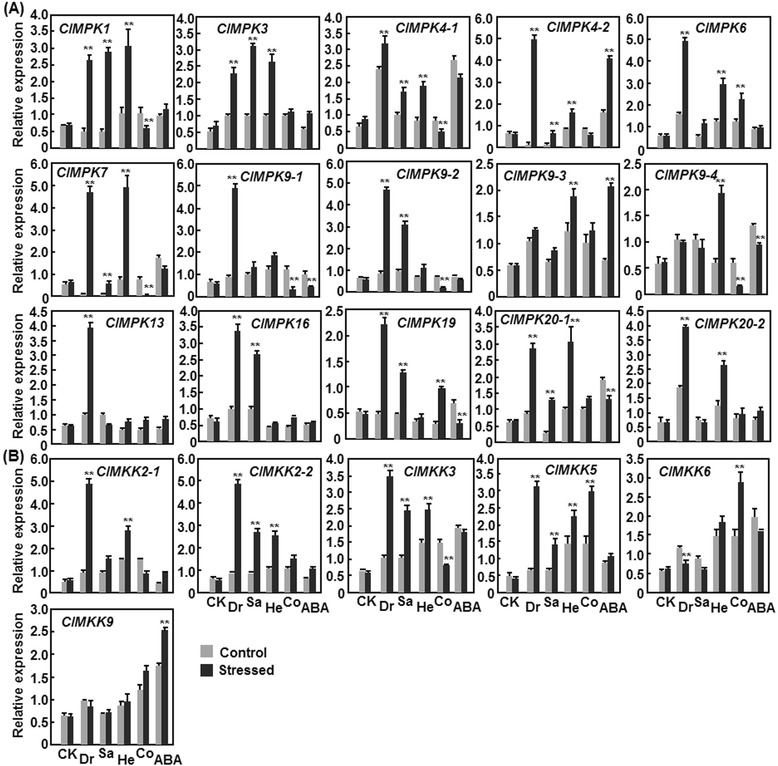
Expression patterns of *ClMPKs* (**a**) and *ClMKKs* (**b**) in response to abiotic stress and ABA. Three-week-old plants were treated by drought (placing on lab bench without water supply), salt (drenching with 200 mM NaCl), heat (42 °C) and cold (4 °C) stress or by foliar spraying with 100 μM ABA and leaf samples were collected at 12 h after treatment. Relative expressions as folds of the actin transcript level are presented as the means ± SD from three independent experiments. ** on the columns indicate significant difference at *p* ≤ 0.05 between the treatments and corresponding controls. CK, control; Dr, drought; Sa, salt; He, heat; Co, cold

It is well known that the MAPK cascades play important roles in abiotic stress responses in plants and some of the components of the MAPK cascades have been characterized as critical regulators of plant responses to drought, salt and temperature stresses [30, 31]. To explore the involvement of the *ClMPK* and *ClMKK* genes in abiotic stress responses, we analyzed by qRT-PCR their expression patterns and changes in expression in response to four stress treatments (drought, salinity, cold and heat) and to the stress hormone abscisic acid (ABA). Generally, the expression levels of 15 *ClMPK* and 6 *ClMKK* genes were altered with distinct patterns in watermelon plants after treatment with drought, salinity, cold and heat stress and most of the *ClMPK* and *ClMKK* genes showed differential expression patterns in response to at least two treatments (Fig. 6). Specifically, 13 *ClMPKs* (*ClMPK1*, *ClMPK3*, *ClMPK4-1*, *ClMPK4-2*, *ClMPK6*, *ClMPK7*, *ClMPK9-1*, *ClMPK9-2*, *ClMPK13*, *ClMPK16*, *ClMPK19*, *ClMPK20-1* and *ClMPK20-2*) and four *ClMKKs* (*ClMKK2-1*, *ClMKK2-2*, *ClMKK3* and *ClMKK5*) were induced by drought stress (placing on lab bench without water supply) (Fig. 6). Among them, the expression levels of *ClMPK4-2* and *ClMPK7* exhibited >15-fold increases at 12 h after drought stress treatment (Fig. 6a). In response to salt stress (drenching with 200 mM NaCl), the expression of ten *ClMPKs* (*ClMPK1*, *ClMPK3*, *ClMPK4-1*, *ClMPK4-2*, *ClMPK6*, *ClMPK7*, *ClMPK9-2*, *ClMPK16*, *ClMPK19* and *ClMPK20-1*) and three *ClMKKs* (*ClMKK2-2*, *ClMKK3* and *ClMKK5*) was induced at different levels (Fig. 6). Under high temperature (heat treatment at 42 °C) stress condition, the expression of ten *ClMPKs* (*ClMPK1*, *ClMPK3*, *ClMPK4-1*, *ClMPK4-2*, *ClMPK6*, *ClMPK7*, *ClMPK9-3*, *ClMPK9-4*, *ClMPK20-2* and *ClMPK20-2*) and four *ClMKKs* (*ClMKK2-1*, *ClMKK2-2*, *ClMKK3* and *ClMKK5*) was upregulated with different folds of increases over those in the control plants (Fig. 6). Among these heat-inducible *ClMPK* and *ClMKK* genes, the expression levels of *ClMPK7*, *ClMPK9-4* and *ClMPK20-1* showed >3-fold of increases at 12 h after heat treatment (Fig. 6a). Unlike the upregulated expression patterns of most members in the *ClMPK* and *ClMKK* families in response to drought, salt and heat stresses, the expression of *ClMPKs* and *ClMKKs* exhibited diverse patterns under low temperature condition (cold treatment at 4 °C). For example, the expression levels of five *ClMPKs* (*ClMPK6*, *ClMPK13*, *ClMPK16*, *ClMPK19* and *ClMPK20-1*) and three *ClMKKs* (*ClMKK5*, *ClMKK6* and *ClMKK9*) were increased while the expression of seven *ClMPKs* (*ClMPK1*, *ClMPK4-1*, *ClMPK4-2*, *ClMPK7*, *ClMPK9-1*, *ClMPK9-2* and *ClMPK9-4*) and two *ClMKKs* (*ClMKK2-1* and *ClMKK3*) was downregulated at 12 h after cold treatment (Fig. 6). By contrast, the expression of *ClMPK3*, *ClMPK9-3*, *ClMPK20-2* and *ClMKK2-2* was not affected markedly under cold stress condition (Fig. 6). Collectively, some members such as *ClMPK1*, *ClMPK3*, *ClMPK7* and *ClMPK19* in the *ClMPK* family and *ClMKK2-2*, *ClMKK3* and *ClMKK5* in the *ClMKK* family exhibited upregulated expression under three stress treatments (Fig. 6), indicating that these *ClMPK* and *ClMKK* genes may have functions in response to multiple stresses. Interestingly, the expression of *ClMPK7* was repressed in cold stress but was induced significantly in heat stress (Fig. 6a), suggesting that ClMPK7 may play opposite roles in cold and heat stress responses via different MAPK cascades. Furthermore, the expression of the paralog pair *ClMPK4-1*/*ClMPK4-2* showed similar patterns while the paralog pairs *ClMPK9-1*/*ClMPK9-4*, *ClMPK20-1*/*ClMPK20-2* and *ClMKK2-1*/*ClMKK2-2* exhibited distinct patterns in response to different abiotic stress treatments (Fig. 6).

It is well known that ABA and the ABA-mediated signaling pathway play central roles in abiotic stress response in plants through triggering major changes in gene expression and adaptive physiological responses [30, 32, 33]. Recently, MAPK cascades have been demonstrated to be implicated in ABA signaling that is involved in abiotic stress response [30]. Thus, we further analyzed the expression patterns of the *ClMPK* and *ClMAKK* genes in response to exogenous ABA. As shown in Fig. 6, the expression levels of three *ClMPKs* (*ClMPK3*, *ClMPK4-2* and *ClMPK9-3*) and three *ClMKKs* (*ClMKK2-1*, *ClMKK2-2* and *ClMKK9*) were increased while the expression levels of six *ClMPKs* (*ClMPK4-1*, *ClMPK7*, *ClMPK9-1*, *ClMPK9-4*, *ClMPK19* and *ClMPK20-1*) and one *ClMKK* (*ClMKK6*) were decreased after ABA treatment. By contrast, the expression of *ClMPK1*, *ClMPK6*, *ClMPK9-2*, *ClMPK13*, *ClMPK16*, *ClMPK20-2*, *ClMKK3* and *ClMKK5* was not affected by exogenous ABA (Fig. 6). Notably, the expression of some members such as *ClMPK4-1*, *ClMPK7*, *ClMPK19* and *ClMPAK20-1* in the *ClMPK* family and *ClMKK3* in the *ClMKK* family showed distinct and even opposite patterns in response to abiotic stress and exogenous ABA (Fig. 6). This does not imply that ABA and its signaling are not involved in the response to abiotic stresses that regulate the expression of these *ClMPKs* and *ClMKKs* as the activity and function of the MAPK cascades depend largely on the phosphorylation status of the components.

#### Expression patterns in response to pathogen infection

**Fig. 7:**
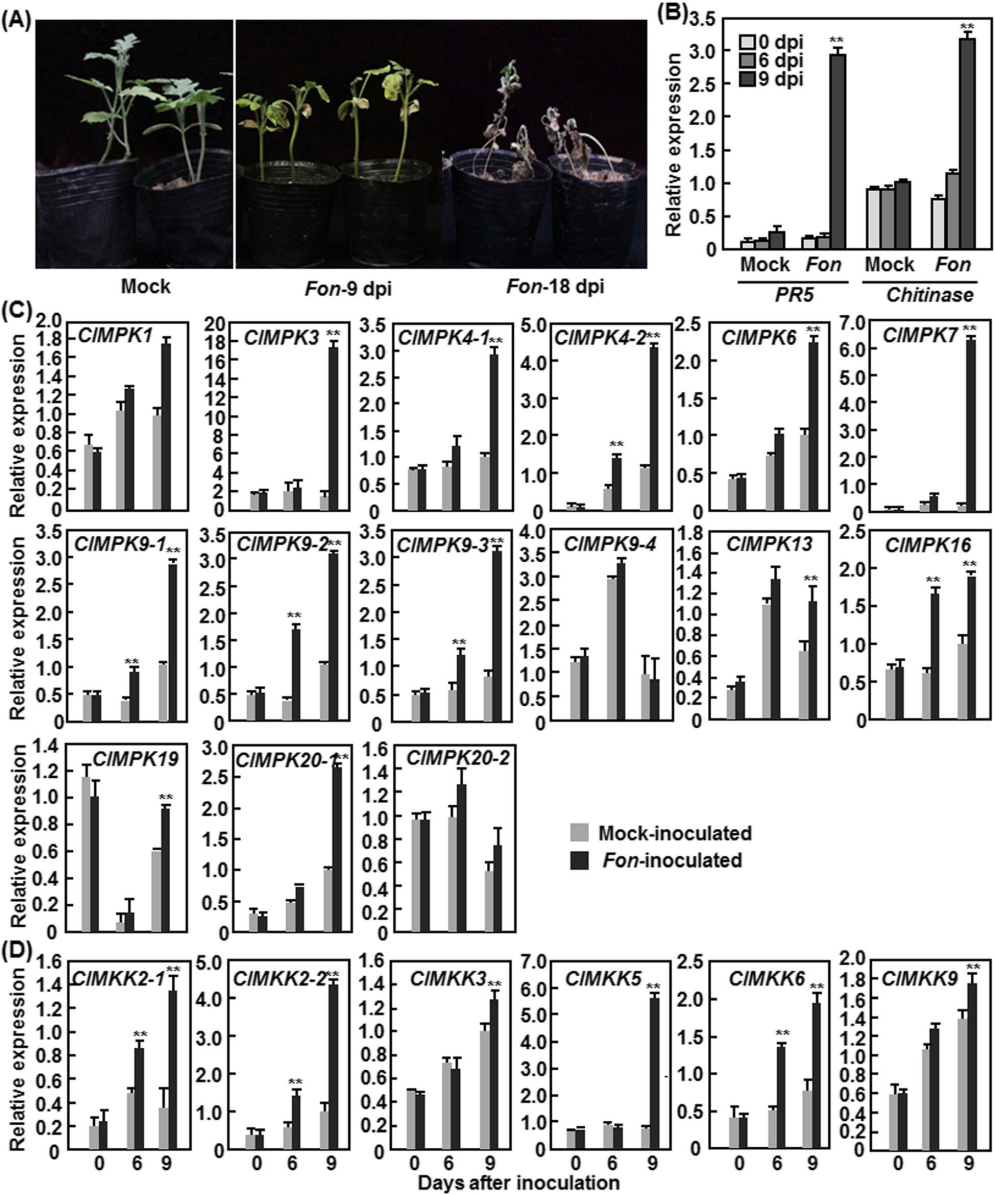
Expression patterns of *ClMPKs* (**a**) and *ClMKKs* (**b**) in response to *Fusarium oxysporum* f. sp. *niveum*. Two-week-old plants were inoculated by dipping the roots in conidia suspension (1 × 10^7^ conidia/mL) of *F. oxysporum* f. sp. *niveum* or in sterilized water as mock-inoculated controls. Disease progress was monitored (**a**) and leaf samples were collected at indicated time points for analyzing the expression of defense marker genes (**b**) and the *ClMPK* (**c**) and *ClMKK* (**d**) genes. Relative expressions as folds of the actin transcript level are presented as the means ± SD from three independent experiments. ** on the columns indicate significant differences at *p* ≤ 0.05 between the pathogen- and mock-inoculated plants

The functions of MAPK cascades in plants disease resistance have been well documented both in the model plants and crops [1, 6]. To explore the involvement of *ClMPKs* and *ClMKKs* in disease resistance, we analyzed their expression patterns in watermelon plants after infection with *Fusarium oxypsorum* f. sp. *niveum* (*Fon*), the most important soilborne fungal pathogen causing Fusarium wilt disease limiting watermelon production in many areas of the world [34, 35]. To do this, we inoculated the two-week-old plants with *Fon* spore suspension and monitored the disease progress over a period of 3 weeks. In our 4 independent experiments, the average of the disease incidence was approximately 90 %. Typical symptom of Fusarium wilt disease, showing wilted leaves, was observed at 9 days after inoculation (dpi) in *Fon*-inoculated plants but not in the mock-inoculated plants and most of the *Fon*-inoculated plants died at 18 dpi (Fig. 7a). To examine the defense response in watermelon plants after infection by *Fon*, we analyzed and compared the expression patterns of two defense-related genes, *ClPR5* and *Chitinase*, in the *Fon*-inoculated and mock-inoculated plants. As shown in Fig. 7b, the expression levels of *ClPR5* and *Chitinase* in the *Fon*-inoculated plants were comparable to those in the mock-inoculated plants at 6 dpi; however, the levels in the *Fon*-inoculated plants were significantly increased at 9 dpi, showing approximately 8- and 3-fold of increases over those in the mock-inoculated plants (Fig. 7b), indicating an activation of defense response in the *Fon*-inoculated plants. We then analyzed the expression patterns of *ClMPKs* and *ClMKKs* in response to *Fon* using the samples collected form the *Fon*- and mock-inoculated plants, which were verified by monitoring of disease progress and expression of defense-related genes (Fig. 7a and b). As shown in Fig. 7c and d, the expression levels of 12 *ClMPKs* (*ClMPK1*, *ClMPK3*, *ClMPK4-1*, *ClMPK4-2*, *ClMPK6*, *ClMPK7*, *ClMPK9-1*, *ClMPK9-2*, *ClMPK9-3*, *ClMPK13*, *ClMPK16* and *ClMPK20-1*) and four *ClMKKs* (*ClMKK2-1*, *ClMKK2-2*, *ClMKK5* and *ClMKK6*) were altered with distinct patterns in watermelon plants after *Fon* infection, indicating that these *ClMPKs* and *ClMKKs* are *Fon*-inducible. However, the expression of *ClMPK9-4*, *ClMPK19*, *ClMPK20-2*, *ClMKK3* and *ClMKK9* was not affected significantly by *Fon* infection. Furthermore, the expression of these *Fon*-inducible *ClMPKs* and *ClMKKs* exhibited distinct patterns in terms of time-course and magnitude of the *Fon*-induced expression. For example, the expression levels of *ClMPK9-2*, *ClMPK16* and *ClMKK6* were increased significantly at 6 dpi and showed further increases at 9 dpi while the expression levels of other *Fon*-inducible *ClMPKs* and *ClMKKs* were only increased significantly at 9 dpi (Fig. 7c and d). At 9 dpi, the expression levels of *ClMPK2-1*, *ClMPK2-2*, *ClMPK4-1*, *ClMPK4-2* and *ClMPK9-3* exhibited >3-fold and the expression levels of *ClMPK3*, *ClMPK7* and *ClMKK5* showed >6-fold of increases over those in the mock-inoculated plants (Fig. 7c and d).

### Functions of *ClMPK1*, *ClMPK4-2*, *ClMPK7*, *ClMPK6* and *ClMKK2-2* in disease resistance

**Fig. 8:**
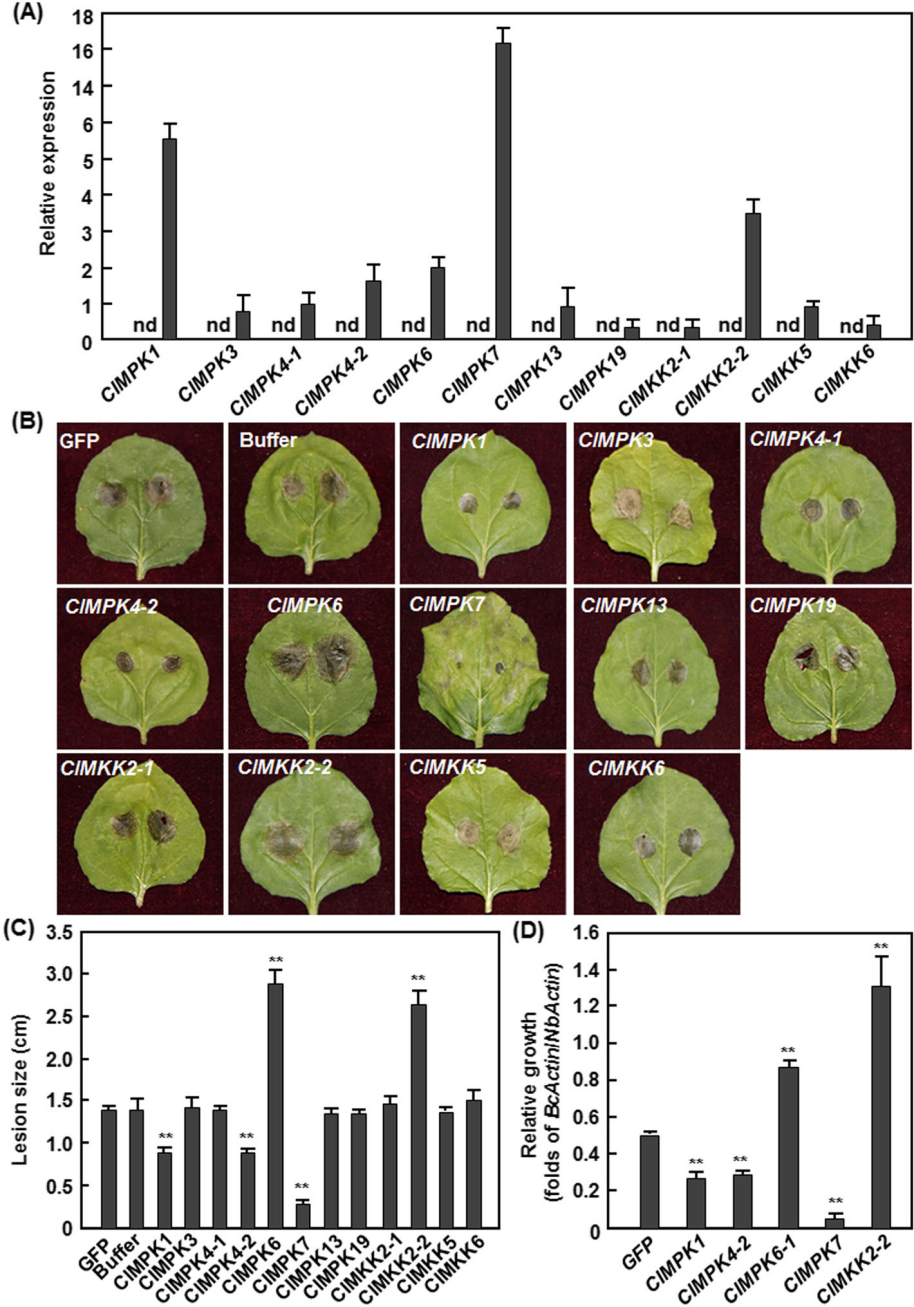
Disease phenotype in *ClMPK-* and *ClMKK*-transiently expressed *N. benthamiana* leaves after inoculation with *B. cinerea*. Agrobacteria harboring different constructs containing *ClMPKs*, *ClMKKs* or eGFP (a negative control) or similar volume of buffer (a negative control) were infiltrated into leaves of 4-week-old *N. benthamiana* plants and the agroinfiltrated leaves were collected for analyzing the expression of *ClMPKs* and *ClMKKs* and for disease assays with *B. cinerea*. **a** Expression levels of selected *ClMPKs* and *ClMKKs* in agroinfiltrated leaves. Leaf sample were collected at 24 h after agroinfiltration and relative expressions as folds of the actin transcript level are presented as the means ± SD from three independent experiments. nd, expression of the *ClMPKs* and *ClMKKs* in eGFP-infiltrated leaves was not detectable. **b** Disease phenotype and **c** lesion sizes on detached leaves and **d** fungal growth in the inoculated leaves. The agroinfiltrated leaves were detached at 2 days after agroinfiltration and disease assays were performed by dropping 5 μL of spore suspension (1 × 10^5^ spores/mL). Photos were taken and lesion sizes were recorded at 4 days after inoculation. Fungal growth in inoculated leaves was assumed by analyzing the transcripts of *BcActin* gene by qRT-PCR using *NbActin* as an internal control at 4 days after inoculation. Data presented in **c** and **d** are the means ± SD from three independent experiments and ** on the columns indicate significant difference at *p* ≤ 0.05 between ClMPK/ClMKK- and eGFP-infiltrated plants

Due to the unavailability of routine transformation of watermelon, we therefore performed functional analyses through ectopic transient expression in *N. benthamiana* to further investigate the functions of *ClMPKs* and *ClMKKs* in disease resistance. To this end, 9 *ClMPKs* (*ClMPK1*, *ClMPK3*, *ClMPK4-1*, *ClMPK4-2*, *ClMPK6*, *ClMPK7*, *ClMPK9-2*, *ClMPK13* and *ClMPK19*) and 5 *ClMKKs* (*ClMKK2-1*, *ClMKK2-2*, *ClMKK5* and *ClMKK6* and *ClMKK9*) were transiently expressed in *N. benthamiana* via agroinfiltration. qRT-PCR analysis with samples collected at 24 h after agroinfiltration indicated that most of the selected *ClMPKs* and *ClMKKs* expressed normally in *N. benthiamina* and their expression levels, shown as folds of the level of a *NbActin* gene, varied greatly in individual *ClMPK*- or *ClMKK*-infiltrated leaves while no transcript for the selected *ClMPKs* and *ClMKKs* was detected in eGFP-infiltrated leaves (Fig. 8a). The expression levels of *ClMPK7*, *ClMPK1* and *ClMKK2-2* were approximately 16-, 5.5- and 3.4-fold, and the expression levels of the remaining selected *ClMPKs* and *ClMKKs* were about 0.3–2.1-fold of the *NbActin* gene (Fig. 8a). Unfortunately, we were unable to detect the expression of *ClMPK9-2* and *ClMKK9* in *N. benthamiana* and thus we did not perform further experiments on these two genes. At 48 h after agroinfiltration for transient expression, the agroinfiltrated leaves were collected for disease assays by dropping spore suspension of *B. cinerea* on both sides of the leaves. Disease phenotyping at 3 day after inoculation revealed that the *B. cinerea*-caused lesions on *ClMPK3*-, *ClMPK4-1*-, *ClMPK13*-, *ClMPK19*-, *ClMKK2-1*-, *ClMKK5*- or *ClMKK6*-infiltrated leaves were comparable to those on eGFP- or buffer-infiltrated control leaves (Fig. 8b and c), suggesting that transient expression of these *ClMPKs* and *ClMKKs* in *N. benthamiana* did not affect the resistance to *B. cinerea*. By contrast, the *B. cinerea*-caused lesions on *ClMPK1*-, *ClMPK4*-*2*-, and *ClMPK7*-infiltrated leaves were significantly smaller (Fig. 8b), showing 38, 36 and 80 % of decrease in size, respectively (Fig. 8c), while the lesions on *ClMPK6-* and *ClMKK2-2*-infiltrated leaves were markedly larger (Fig. 8b), leading to 103 and 87 % of increase in size, respectively (Fig. 8c), as compared with those on eGFP- or buffer-infiltrated leaves, indicating that transient expression of *ClMPK7*, *ClMPK1*, *ClMPK4-2*, *ClMPK6* or *ClMKK2-2* in *N. benthamiana* affected the resistance to *B. cinerea*. Analysis of the transcript for the *B. cinerea actin* gene *BcActinA* as an indicator of the rate of *in planta* fungal growth indicated that growth of *B. cinerea* in the *ClMPK1*-, *ClMPK4*-*2*-, and *ClMPK7*-infiltrated leaves was significantly lower, showing 52, 50 and 91 % of decrease, respectively; whereas the growth in the *ClMPK6-* and *ClMKK2-2*-infiltrated leaves was markedly higher, resulting in 72 and 160 % of increase, respectively, as compared with those on eGFP-infiltrated control leaves (Fig. 8d).

**Fig. 9:**
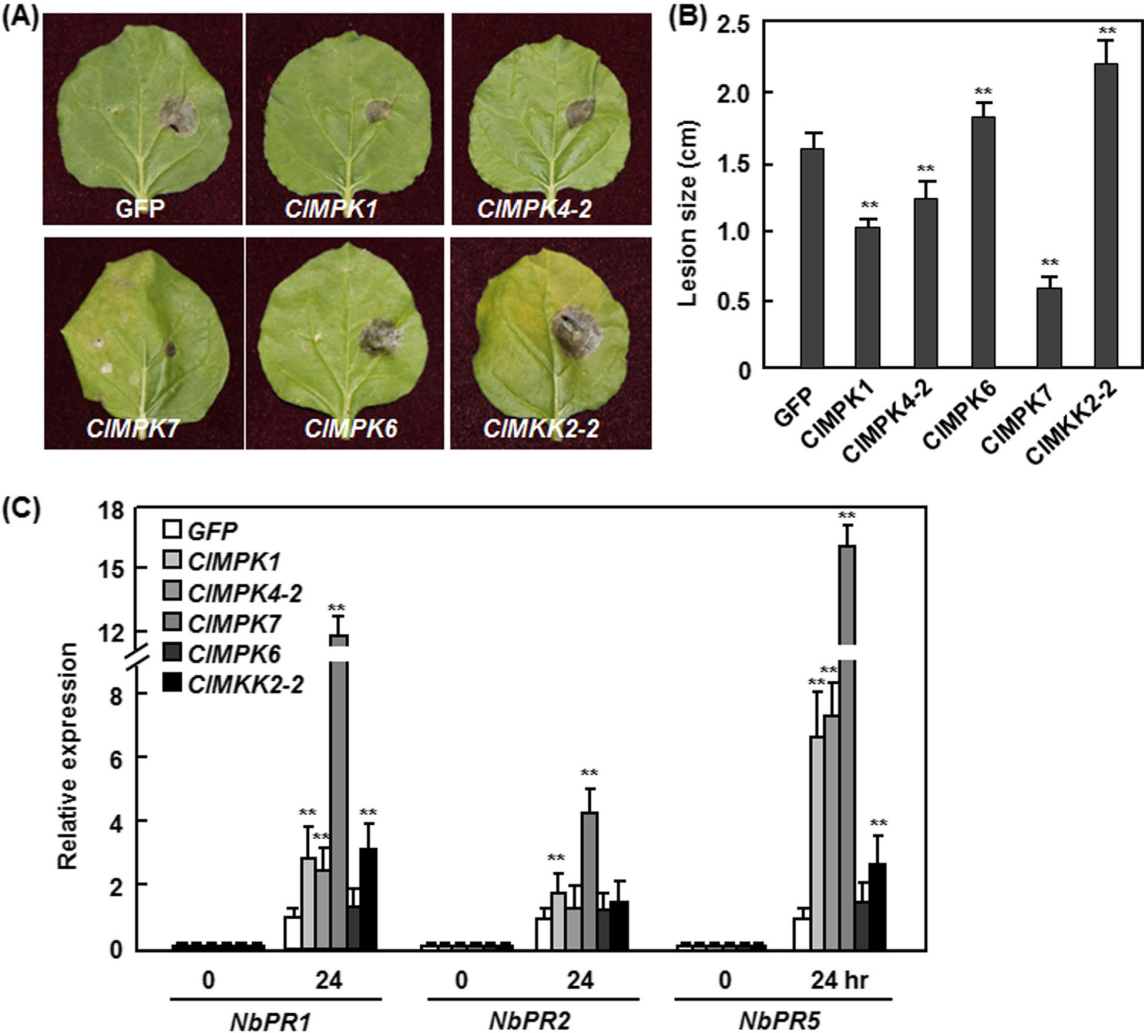
Effects of *ClMPK1*, *ClMPK4-2*, *ClMAK6*, *ClMPK7* and *ClMKK2-2* on systemic resistance to *B. cinerea* and the expression of defense-related genes. Agrobacteria harboring constructs containing *ClMPK1*, *ClMPK4-2*, *ClMPK6*, *ClMPK7*, *ClMKK2-2* or eGFP (a negative control) were infiltrated into one half of 4-week-old *N. benthamiana* leaves and the agroinfiltrated leaves were collected for disease assays with *B. cinerea*. Disease assays were performed by dropping 5 μL of spore suspension (1 × 10^5^ spores/mL) onto the opposite half of the leaves at 2 days after agroinfiltration. Photos for disease phenotype **a** were taken and lesion sizes **b** were measured at 4 days after inoculation. **c** Expression of defense-related genes in *ClMPK1*-, *ClMPK4-2*-, *ClMPK6*-, *ClMPK7*- and *ClMKK2-2*-transiently expressed leaves. Leaf samples were collected at 24 h after agroinfiltration and relative expressions are shown as folds of the actin transcript level. Data presented in **b** and **c** are the means ± SD from three independent experiments and ** on the columns indicate significant difference at *p* ≤ 0.05 between *ClMPK*/*ClMKK*- and eGFP-infiltrated plants

It was previously shown that overexpression of the Arabidopsis *AtMKK7* leads to activation of systemic acquired resistance [36], a form of inducible immune responses in plants [37]. We therefore examined whether transient expression of *ClMPK1*, *ClMPK4-2*, *ClMPK7*, *ClMPK6* or *ClMKK2-2* in *N. benthamiana* affect the resistance of distal tissues to *B. cinerea*. For this purpose, agrobacteria carrying the constructs containing *ClMPK1*, *ClMPK4-2*, *ClMPK7*, *ClMPK6* or *ClMKK2-2* were infiltrated into one half of the leaves and disease assays with *B. cinerea* were performed on the opposite half of the agroinfiltrated leaves at 2 days after agroinfiltration. Disease phenotyping at 3 day after inoculation revealed that the *B. cinerea*-caused lesions on the opposite half of the *ClMPK1*-, *ClMPK4*-*2*-, and *ClMPK7*-infiltrated leaves were significantly smaller (Fig. 9a), showing 38, 25 and 64 % of decrease in size, respectively (Fig. 9b), while the lesions on the opposite half of the *ClMPK6-* and *ClMKK2-2*-infiltrated leaves were markedly larger (Fig. 9a), resulting in 12 and 35 % of increase in size, respectively (Fig. 9b), as compared with those on eGFP-infiltrated control leaves.

To explore the possible molecular mechanisms for the actions of *ClMPK1*, *ClMPK4-2*, *ClMPK7*, *ClMPK6* and *ClMKK2-2* in disease resistance, we analyzed whether transient expression of these *ClMPKs* and *ClMKKs* affected the expression of defense-related genes in *N. benthamiana*. The expression levels of *NbPR1*, *NbPR2* and *NbPR5*, three defense-related genes [38], in *ClMPK1-*, *ClMPK4-2-*, *ClMPK7-*, *ClMPK6-* or *ClMKK2-2*-transiently expressed leaves were analyzed and compared with those in eGFP-infiltrated leaves. As shown in Fig. 9c, no expression of the tested defense-related genes was detected at 0 h after agroinfiltration; however, increased expression of these genes at 24 h after agroinfiltration in *ClMPK1-*, *ClMPK4-2-*, *ClMPK7-*, or *ClMKK2-2*-transiently expressed leaves was observed. The expression levels of *NbPR1* and *NbPR5* were significantly increased at 24 h after agroinfiltration in *ClMPK1-*, *ClMPK4-2-* or *ClMPK7-*transiently expressed leaves, leading to 1.3 ~ 10.8 folds for *NbPR1* and 5.5 ~ 15.5 folds for *NbPR5* over those in the eGFP-infiltrated leaves (Fig. 9c). Increased expression of *NbPR2* in *ClMPK1-* or *ClMPK7-*transiently expressed leaves and of *NbPR1* and *NbPR5* in *ClMKK2-2-*transiently expressed leaves were also observed (Fig. 9c). However, the expression levels of *NbPR1*, *NbPR2* and *NbPR5* in *ClMPK6*-transiently expressed leaves were comparable to those in eGFP-infiltrated leaves (Fig. 9c).

### Function of *ClMPK7* in hypersensitive response-like cell death

**Fig. 10:**
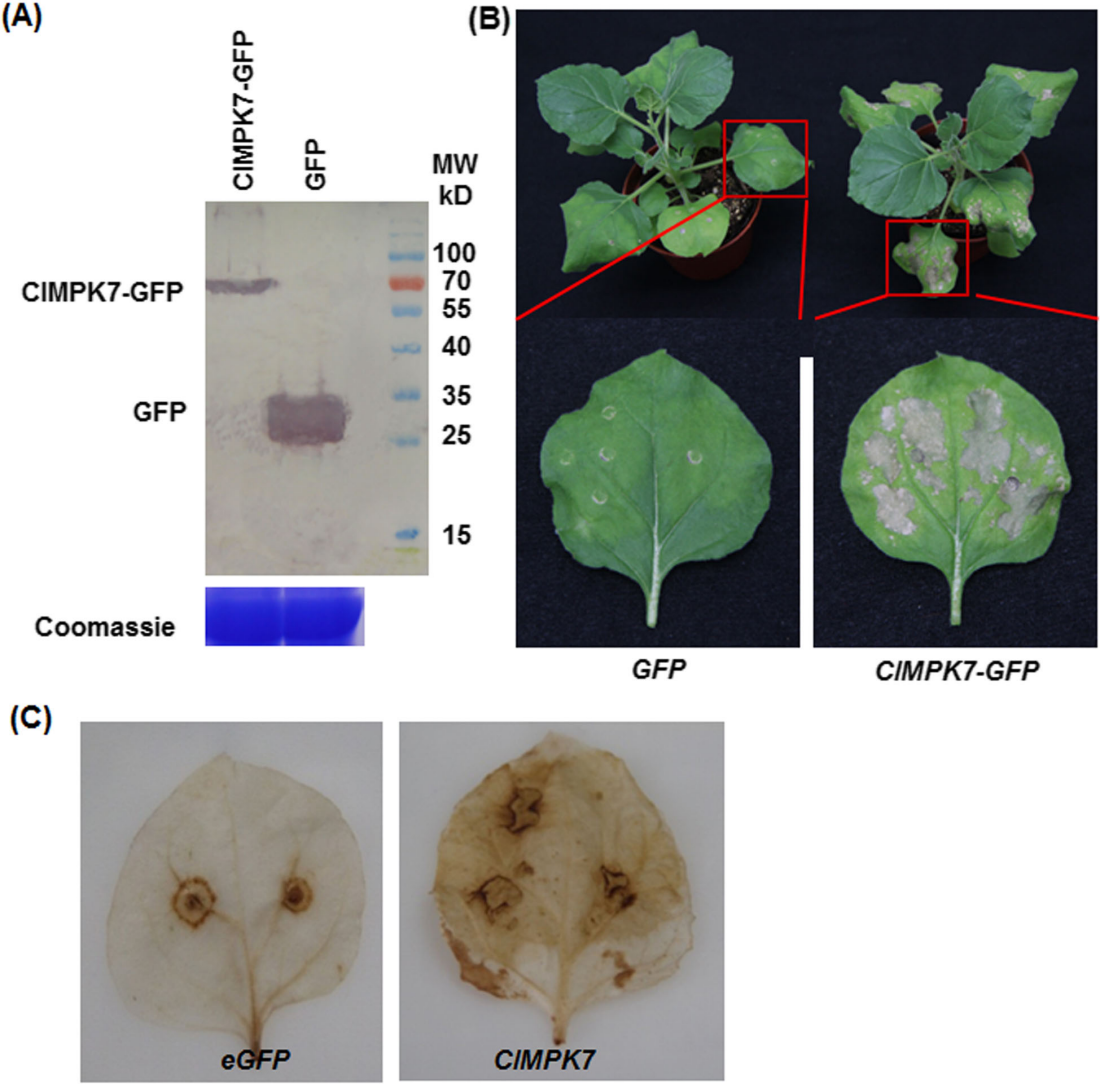
Transient expression of *ClMPK7* triggered HR-like cell death and accumulation of H_2_O_2_. Agrobacteria harboring constructs containing *ClMPK7* or eGFP (a negative control) were infiltrated into leaves of 4-week-old *N. benthamiana* plants. **a** Detection of ClMPK7 in *ClMPK7*-transiently expressed leaves. Leaf samples were harvested at 24 h after agroinfiltration and total soluble proteins were extracted. Proteins were separated by SDS–PAGE and analyzed by immunoblotting using a GFP-specific antibody. Total proteins showing equal loading were examined by Coomassie staining. **b** HR-like cell death in *ClMPK7*-transiently expressed leaves. Photos were taken at 7 days after agroinfiltration and representative leaves showing HR-like cell death (large necrotic lesions) were particularly presented. **c** Accumulation of H_2_O_2_. Leaf samples were collected at 24 h after agroinfiltration and H_2_O_2_ accumulation was detected by DAB staining. Repeated experiments showed similar results

During our transient expression-based functional analysis of the selected *ClMPKs* and *ClMPKs* in disease resistance, we noted that the *ClMPK7*-transiently expressed leaves exhibited typical hypersensitive response (HR)-like cell death while other selected *ClMPKs* or *ClMKKs*-transiently expressed leaves did not (Fig. 9a), indicating an involvement of *ClMPK7* in HR-like cell death. Therefore, several experiments were conducted to confirm the possible function of *ClMPK7* in HR-like cell death. At 24 h after agroinfiltration, significant accumulation of the ClMPK7 protein as a ClMPK7-GFP fusion was clearly detected in ClMPK7-GFP-infiltrated leaves while only GFP was detected in eGFP-infiltrated leaves (Fig. 10a). In ClMPK7-GFP-infiltrated leaves, typical HR-like cell death as small necrotic lesions in the infiltration area was observed at 24 h and these necrotic lesions enlarged with times, forming large necrotic area at 7 days after agroinfiltration (Fig. 10b). Only slight cell death at the infiltration site was observed in eGFP-infiltrated leaves, probably due to wounding during infiltration process (Fig. 10b). Furthermore, significant accumulation of H_2_O_2_, as detected by 3, 3-diaminobenzidine (DAB) staining, was observed in ClMPK7-GFP-infiltrated leaves, not only at the infiltration site but also in the tissues surrounding the infiltration sites, while the H_2_O_2_ accumulation was only seen at the infiltration sites (Fig. 10c). These data indicate that ClMPK7 plays a role in HR-like cell death probably through modulating the generation of H_2_O_2_.

## Discussion

The MAPK cascades are one of the major pathways that play critical roles in growth and development as well as in stress responses. The MPKs and MKKs, the two last components in the MAPK cascades, are represented as multigene families, which have been studied in detail at the genome-wide level in a number of plants species. Our genome-wide survey identified 15 ClMPKs and 6 ClMKKs in watermelon, which can be classified into four distinct groups. Data from our detailed studies on some selected members of the ClMPK and ClMKK families for their protein-protein interaction relationships, expression patterns in different tissues and in response to abiotic and biotic stress, and possible functions in disease resistance provide the first line of evidence for the biological functions of the ClMPK and ClMKK families in watermelon.

The function and activity of components in the MAPK cascades depend on their direct physical interactions. In the present study, complicated interaction relationships and specificity between ClMPKs and ClMKKs were observed. For examples, ClMPK4-1 interacted with two ClMKKs (ClMKK2-1 and ClMKK6) while ClMKK2-1 interacted with three ClMPKs (ClMPK4-1, ClMPK4-2 and ClMPK13 (Fig. 4). ClMKK2-2 interacted specifically with ClMPK1 and vice versa (Fig. 4). ClMKK2-1 and ClMKK2-2, which have high levels of sequence similarity (Fig. 2), interacted with different ClMPKs (Fig. 4). The complicated interaction relationships and specificity between ClMPKs and ClMKKs indicate that they may integrate into divergent signaling pathways and determine specific biological functions [2, 39].

The CD domain, which is thought to be involved in interacting with upstream MKKs [40], seems not the sole domain responsible for protein-protein interaction between ClMPKs and ClMKKs. It is reasonable that ClMPK4-1, ClMPK4-2, ClMPK6 and ClMPK13 interacted differentially with corresponding ClMKKs (Fig. 4), as all these four ClMPKs contain the CD domain. Surprisingly, however, ClMPK1 and ClMPK7, which do not have the CD domain, interacted with ClMKK2-2 and ClMKK5, respectively (Fig. 4). This is similar to the previous observations that *B. distachyon* BdMPK7-1/14/17 and canola BnaMPK9/19/20, all lacking the CD domain, could interact with upstream corresponding MKKs [17, 22]. Thus, it is likely that other domains/motifs in ClMPKs may be involved in determination of the interaction with upstream ClMKKs.

It was previously demonstrated that the Arabidopsis AtMKK1/AtMKK2 interact with AtMPK4, forming AtMKK1/AtMKK2-AtMPK4 cascade, while AtMKK4/AtMKK5 interact with both of AtMPK3/AtMPK6, leading to AtMKK4/AtMKK5-AtMPK3/AtMPK6 cascades [41–45]. Similar interactions between ClMPKs and ClMKKs were observed. For example, ClMKK2-1, closely related to AtMKK1/AtMKK2 (Fig. 2), interacted significantly with ClMPK4-1 and ClMPK4-2, two ClMPKs that are phylogenetically clustered with AtMPK4 (Fig. 2), whereas ClMKK5, a putative ortholog of AtMKK4 and AtMKK5, interacted strongly with ClMPK6, a ClMPK with high level of similarity to AtMPK6. The fact that ClMKK6 interacted with ClMPK2-1, ClMPK2-2 and ClMPK13 is similar to the Arabidopsis AtMKK6, which can interact and phosphorylate AtMPK4 and AtMPK13 [46–48]. Interestingly, ClMKK2-1 and ClMKK6 interacted with the same ClMPKs including ClMPK4-1, ClMPK4-2 and ClMPK13 (Fig. 4). Collectively, it is likely that ClMKK2-1/ClMKK6-ClMPK4-1/ClMPK4-2/ClMPK13 and ClMKK5-ClMPK6 in watermelon may constitute separate MAPK cascades. However, like those in Arabidopsis and rice [43, 49], further comprehensive analysis of protein-protein interactions among ClMKKs and ClMPKs will be helpful to establish the MAPK cascades and their signaling networks.

Although activity of the MAPK cascades can be regulated at both transcriptional and post-translational levels, transcriptional regulation of expression of MPK and MKK genes was reported previously in a range of plants. It is generally accepted that a gene expressed abundantly in a tissue or during a developmental stage or increasingly under a stress condition may imply its function related to developmental and stress response. In this regard, the expression patterns of *ClMPKs* and *ClMKKs* in different tissues or in response to biotic and abiotic stresses may indicate the biological functions of and the possible relationships between ClMPKs and ClMKKs in watermelon. The expression of members in the putative ClMKK2-1/ClMKK6-ClMPK4-1/ClMPK4-2/ClMPK13 and ClMKK5-ClMPK6 cascades showed similar upregulated patterns in response to *Fon* infection (Fig. 7). Similarly, the expression of *ClMPK6* and *ClMKK5* in the ClMKK5-ClMPK6 cascade was synchronously upregulated by drought, heat and cold stresses (Fig. 6). However, different expression patterns of the members in these two putative MAPK cascades in different tissues and upon abiotic and biotic stress treatments were also noted. For example, salt stress induced the expression of *ClMPK4-1* and *ClMPK4-2* but did not affect the expression of *ClMKK2-1* and *ClMKK6* (Fig. 6). The difference in expression patterns of the members in a putative MPAK cascade may be explained by the nature that the biochemical function of the MAPK cascades is mainly determined by the phosphorylation status of the components in the cascades or that other components exist to form unknown cascades under specific growth and stress conditions. Another, different expression patterns of some paralog pairs were observed. For example, the expression of the paralog pair ClMPK4-1/ClMPK4-2 showed similar patterns while the paralog pairs ClMPK9-1/ClMPK9-4, ClMPK20-1/ClMPK20-2 and ClMKK2-1/ClMKK2-2 exhibited distinct expression patterns in response to different abiotic stress treatments (Fig. 6). This is similar to the results observed in the cotton MPK family under different abiotic stress treatments [20]. It is thus likely that some members of the ClMPK and ClMKK families may retain the functional conservation while others evolve to possess divergent functions to cope with different environmental challenges.

Our expression analyses revealed that the *ClMPK* and *ClMKK* families respond with different patterns to *Fon* infection and that *ClMPK3*, *ClMPK7* and *ClMKK5* were significantly induced by *Fon* (Fig. 7), indicating their possible involvements in the activation of defense response in watermelon to *Fon*. Further transient expression-based functional analyses demonstrated that *ClMPK1*, *ClMPK4*-*2* and *ClMPK7* positively but *ClMPK6* and *ClMKK2-2* negatively regulate the resistance to *B. cinerea* when transiently expressed in *N. benthamiana* (Fig. 8). The fact that transient expression of *ClMPK1*, *ClMPK4-2*, *ClMPK7*, *ClMPK6* and *ClMKK2-2* in *N. benthamiana* affected the resistance of distal tissues to *B. cinerea* not only confirmed their functions in disease resistance but also suggest systemic effects on activation of defense response (Fig. 9). *ClMPK1* and *ClMPK7* belong to group C and phylogenetically related to Arabidopsis *AtMPK1*, *AtMPK2*, *AtMPK7* and *AtMPK14* (Fig. 2). In the present study, we found that the expression of *ClMPK7* was induced by several abiotic stresses and by *Fon* (Figs. 6 and 7) and transient expression of *ClMPK7* in *N. benthamiana* resulted in increased resistance to *B. cinerea* (Figs. 8 and 9). This is consistent with the observations that the Arabidopsis AtMPK7, as a component of the AtMKK3-AtMPK7 cascade, was found to play a role in defense responses against *P. syringae* pv. *tomato* DC3000 while overexpression of cotton *GhMPK7* in *N. benthamiana* conferred an increased resistance to *Colletotrichum nicotianae* [50, 51]. ClMPK4-2 is closely related to Arabidopsis AtMPK4 (Fig. 2a). Expression of *ClMPK4-2* was induced at 6 dpi after infection of *Fon* (Fig. 7) and transient expression in *N. benthamiana* resulted in increased resistance to *B. cinerea* (Figs. 8 and 9). It was previously shown that the Arabidopsis *atmpk4* mutant and tomato *SlMPK4*-silenced plants showed enhanced susceptibility to *Alternaria brassicicola* and *B. cinerea*, respectively [52, 53], whereas overexpression of *BnMPK4* in oilseed rape plants significantly enhances resistance to *Sclerotinia sclerotiorum* and *B. cinerea* [54]. These data demonstrate that plant MPK4 including ClMPK4-2 functions as positive regulators of defense response against necrotrophic fungal pathogens. Another ClMPK that has function in disease resistance to *B. cinerea* is ClMPK6, showing high level of similarity to Arabidopsis AtMPK6 (Fig. 2a), which is well documented as a critical component of the MEKK1–MKK4/MKK5–MPK3/MPK6 cascades regulating immune responses [1, 6, 7]. It was found that activation of AtMPK3 and AtMPK6 impeded the infection of *B. cinerea* [55] although lack of AtMPK6 did not affect the basal resistance to *B. cinerea* [56]. This is somewhat different from our observation in the present study that transient expression of *ClMPK6* in *N. benthamiana* led to reduced resistance to *B. cinerea* (Figs. 8 and 9). ClMKK2-2 is a putative ortholog of Arabidopsis AtMKK1 and AtMKK2 (Fig. 2b) in the AtMKK1/AtMKK2-AtMPK4 cascade, which negatively regulates immunity [45]. However, ClMKK2-2 did not interact with ClMPK4-2 (Fig. 4b), which is closely related to AtMPK4 and AtMPK11, and its function of ClMKK2-2 in resistance to *B. cinerea* differs from that of ClMPK4-2 (Figs. 8 and 9). Thus, it is unlikely that ClMKK2-2 and ClMPK4-2 form a functional MAPK cascade. Although ClMKK2-2 interacted with ClMPK1 (Fig. 4b), they had opposite effects on the resistance to *B. cinerea* when transiently expressed in *N. benthamiana* (Figs. 8 and 9). Whether ClMKK2-2 and ClMPK1 form a true functional MAPK cascade needs to be further examined.

We also found in the present study that transient expression of *ClMPK7* in *N. benthamiana* triggered a HR-like cell death and that ClMPK7-induced HR-like cell death was probably initiated by abnormal ROS accumulation (Fig. 10). This is similar to the previous observations that activation of the tobacco SIPK/Ntf4/WIPK and the Aabidopsis AtMKK4/AtMKK5 cascades actively promotes the generation of ROS, which plays an important role in the signaling for and/or execution of HR cell death [57–59]. Notably, transient expression of *ClMPK7* in *N. benthamiana* resulted in significant HR-like cell death and increased resistance to *B. cinerea* (Figs. 8, 9 and 10), which is consistent with the functions of Arabidopsis AtMKK4, tobacco NtMEK2 and tomato SlMKK2/SlMKK4 in HR-like cell death and enhanced resistance to *B. cinerea* [16, 41, 58]. On the other hand, it is well known that expression of constitutively active forms of MKKs can trigger HR-like cell death in plants [16, 38, 41, 58]. However, we did not observed any HR-like cell death in leaves of *N. benthamiana* plants infiltrated constructs carrying wild type forms of *ClMKK2-1*, *ClMKK2-2*, *ClMKK5* and *ClMKK6* (Fig. 8), indicating that transient expression of the wild type forms of these *ClMKKs* cannot trigger HR-like cell death. This is consistent with the observations that overexpression of wild type forms of tomato SlMKK2 and SlMMK4 and the Arabidopsis AtMKK3 did not induce HR-like cell death or affect disease resistance but overexpression of the constitutively active phosphomimicking forms induced significant HR-like cell death or disease resistance [16, 50]. Therefore, the functions of *ClMKK2-1*, *ClMKK2-2*, *ClMKK5* and *ClMKK6* in HR-like cell death need to be further investigated using the constitutively active phosphomimicking forms.

## Conclusion

To date, little is known about the MPK and MKK families and their possible biological functions in watermelon. In addition to the genome-wide characterization of the ClMPK and ClMKK families in watermelon, the present study demonstrated significant interactions between members of the ClMPK and ClMKK families including putative ClMKK2-1/ClMKK6-ClMPK4-1/ClMPK4-2/ClMPK13 and ClMKK5-ClMPK6 cascades and showed the differential expression patterns for most of the members in the ClMPK and ClMKK families in different tissues and in response to abiotic (e.g. drought, salt, cold and heat treatments) and biotic (e.g. *Fon* infection) stresses. Importantly, we found that ClMPK1 and ClMPK7 in Group C and ClMPK4-2 in Group B positively but ClMPK6 in Group A and ClMKK2-2 in Group A of ClMKKs negatively regulate the resistance to *B. cinerea* when transiently expressed in *N. benthamiana* and that ClMPK7 in Group C functions as a regulator of HR-like cell death. The expression patterns, protein-protein interaction relationship, possible functions in disease resistance and their potential functional Arabidopsis orthologs of the ClMPK and ClMKK families are summarized in Table 2. The present work provides an important foundation to direct future functional studies of the ClMPK and ClMKK families in growth/development and stress responses in watermelon. Further genetic studies in watermelon through overexpression and RNA interference approaches will be critical to elucidate the biological functions and molecular mechanisms of the ClMPKs and ClMKKs.

**Table 2:** Summary on the expression, protein-protein interaction, functions in disease resistance and putative Arabidopsis orthologs for the *ClMPK* and *ClMKK* genes

Genes	Expression patterns								Protein-protein interaction^c^	Functions in disease resistance^d^	Homolog in Arabidopsis
	Tissues^a^			Abiotic stress^b^				Biotic stress^b^			
	Root	Stem	Leaf	Dr	Sa	He	Co	*Fon*			
ClMPK1	+++	+	++	↑	↑	↑	↓	–	ClMKK2-2	Increased	AtMPK1
ClMPK3	+	+++	+	↑	↑	↑	–	↑	Not studied	WT	AtMPK3
ClMPK4-1	+	+	+	↑	↑	↑	↓	↑	ClMKK2-1	WT	AtMPK4
									ClMKK6		
ClMPK4-2	+	+	+	↑	↑	↑	–	↑	ClMKK2-2	Increased	AtMPK4 [52]
									ClMKK6		
ClMPK6	+	+	+	↑	–	↑	↑	↑	ClMKK5	Decreased	AtMPK6 [55, 56]
ClMPK7	+++	+	+	↑	↑	↑	↓	↑	ClMKK5	Increased	AtMPK7 [50, 58]
										HR-like cell death	
ClMPK9-1	+++	+	+++	↑	–	–	↓	↑	Not studied	Not studied	AtMPK9
ClMPK9-2	+	+	++	↑	↑	–	↓	↑	–	Not studied	AtMPK9
ClMPK9-3	+	+	++	–	–	↑	–	↑	Not studied	Not studied	AtMPK9
ClMPK9-4	+	+	+++	–	–	↑	↓	–	Not studied	Not studied	AtMPK9
ClMPK13	++	++	+	↑	–	–	–	↑	ClMKK2-1	WT	AtMPK13
									ClMKK6		
ClMPK16	+	+	+++	↑	↑	–	–	↑	–	Not studied	AtMPK16
ClMPK19	+	+	+++	↑	↑	–	↑	↑	Not studied	WT	AtMPK19
ClMPK20-1	+	+++	+++	↑	↑	↑	–	↑	Not studied	Not studied	AtMPK20
ClMPK20-2	++	+	+++	↑	–	↑	–	–	Not studied	Not studied	AtMPK20
ClMKK2-1	+++	++	+	↑	–	↑	–	↑	ClMPK4-1	WT	AtMKK2
									ClMPK4-2		
									ClMPK13		
ClMKK2-2	+	++	++	↑	↑	↑	–	↑	ClMPK1	Decreased	AtMKK2 [45]
ClMKK3	+	++	+	↑	↑	↑	↓	↑	Not studied	Not studied	AtMKK3
ClMKK5	+	+++	+	↑	↑	↑	↑	↑	ClMPK6	WT	AtMKK5
									ClMPK7		
ClMKK6	+++	+++	+	↓	–	–	↑	↑	ClMPK4-1	WT	AtMKK6
									ClMPK4-2		
									ClMPK13		
ClMKK9	++	++	++	–	–	–	–	↑	Not studied	Not studied	AtMKK9

^a^+ represents the relative expression levels

^b^↑represents significant upregulation; ↓indicates significant downregulation; – indicates no significant change. Dr, drought stress; Sa, salt stress; He, heat stress; Co, cold stress. *Fon*, *Fusarium oxypsorum* f. sp. *niveum*

^c^Putative interacting partners from yeast two hybrid assays are listed.–indicates no interacting partner was identified. Not studied, these ClMPKs or ClMKKs were not examined

^d^Possible functions of the selected ClMPKs and ClMKKs was examined using transient expression-based functional analysis in *N. benthamiana*. Increased, increased resistance against *B. cinerea* when transiently expressed in *N. benthamiana*; Decreased, decreased resistance against *B. cinerea* when transiently expressed in *N. benthamiana*. WT, wild-type phenotype

## Methods

### Plant growth and treatments

Watermelon (*Citrullus lanatus*) cv. Zaojia was used for all experiments. Plants were grown in a mixture of perlite: vermiculite: plant ash (1:6:2) in a growth room under fluorescent light (200 μE m^2^ s^−1^) at 22–24 °C with 60 % relative humidity and a 14 h light/10 h dark cycle and three-week-old plants were used unless indicated otherwise. For analysis of tissue-specific expression, leaf, stem and root samples were collected and stored at −80 °C till use. For ABA treatment, plants were treated by spraying with 100 μM ABA or with equal volume of solution containing only 0.1 % ethanol and 0.02 % Tween-20 as a control. For cold stress treatment, plants were transferred to a growth chamber at 4 °C or kept at 25 °C as a control for 24 h. For heat treatment, plants were transferred to a growth chamber at 42 °C or kept at 25 °C as a control for 24 h. For drought stress treatment, plants were put on lab blench without water supply or on water-saturated filter papers as a control for 12 h. For salt stress treatment, plants were irrigated with 200 mM NaCl solution or water as a control at 25 °C. For analysis of gene expression in response to *Fon* infection, inoculation was performed according to a previously reported method [60]. Briefly, conidia were collected from 10-day-old culture of *Fon* race 1 and adjusted to 1 × 10^6^ conidia/mL. Two-week-old plants were carefully uprooted, washed in tap water and then roots of the plants were dipped for 30 s in the conidial suspension or in distilled sterilized water as mock-inoculated controls. The inoculated plants were carefully replanted in soil and allowed to grow in the same growth room as described above. Leaf samples were collected at indicated time points after the treatments and stored at −80 °C till use.

### Characterization and nomenclature of the watermelon ClMPK and ClMKK genes

The Arabidopsis AtMPKs and AtMKKs were used as queries to search for putative MPK and MKK proteins against the watermelon genome database at http://www.icugi.org/. The obtained nucleotide and protein sequences were examined by domain analysis programs PFAM (http://pfam.sanger.ac.uk/) and SMART (http://smart.emblheidelberg.de/) with the default cutoff parameters. The isoelectric points and molecular weights were predicted on the ExPASy Proteomics Server (http://expasy.org/). Sequence alignment was carried out by the ClustalX program. Phylogenetic tree was constructed using the neighbor-joining method of the MEGA6 program with the p-distance and complete deletion option parameters. The reliability of the obtained trees was tested using a bootstrapping method with 1000 replicates.

### Cloning of the *ClMPK* and *ClMKK* genes

Total RNA was extracted by Trizol regent and treated with RNase-free DNase (TaKaRa, Dalian, China) according to the manufacturer’s instructions. First-strand cDNA was synthesized using AMV reverse transcriptase (Takara, Dalian, China) with oligo d(T) primer according to the manufacturer’s instructions. The obtained cDNAs were used for qRT-PCR and cloning. The coding sequences for *ClMPKs* and *ClMKKs* were amplified using gene-specific primers (Additional file 1: Table S1) designed based on the predicated cDNAs and cloned into pMD19-T vector via T/A cloning, yielding pMD19-ClMPKs or pMD19-ClMKKs. After confirmation by sequencing, these pMD19-ClMPKs or pMD19-ClMKKs plasmids were used as templates to amplify the target genes for further experiments.

### Yeast two-hybrid assays

Putative interactions between ClMPKs and ClMKKs were examined using the Matchmaker Gold Yeast Two-Hybrid System according to the manufacturer’s instructions (Clontech, Mountain View, CA, USA). The coding sequences of ClMPKs and ClMKKs were amplified using gene-specific primers (Additional file 1: Table S1) from pMD19-ClMPKs or pMD19-ClMKKs and cloned into pGADT7 and pGBKT7 vectors. The resultant plasmids were transformed into yeast strains Y2HGold and confirmed by colony PCR. The transformed yeasts were cultivated on SD/Trp^−^His^−^ medium for 3 days at 30 °C, followed by addition of X-α-gal (5-Bromo-4chloro-3-indolyl-a-D-galactopyranoside). Interactions between ClMPKs and ClMKKs were evaluated according to the growth situation of the transformed yeast cells on the SD/Trp^−^His^−^ medium and the production of blue pigments after the addition of X-α-Gal. Co-transformation of pGBKT7-53 or pGBKT7-Lam and pGADT7-T were as positive and negative controls, respectively.

### Transient expression in *N. benthamiana* and disease assays

The coding sequences of the selected *ClMPKs* and *ClMKKs* were amplified using gene-specific primers (Additional file 1: Table S1) from the corresponding pMD19-ClMPKs or pMD19-ClMKKs plasmids and cloned into pFGC-Egfp at different restriction enzyme sites, yielding pFGC-ClMPKs or pFGC-ClMKKs. The recombinant plasmids pFGC-ClMPKs or pFGC-ClMKKs and the empty vector pFGC-Egfp were introduced into *Agrobacterium tumefaciens* strain GV3101 by electroporation using GENE PULSER II Electroporation System (Bio-Rad Laboratories, Hercules, CA, USA). Agrobacteria carrying pFGC-ClMPKs, pFGC-ClMKKs or pFGC-Egfp were grown in YEP medium (50 μg/ml rifampicin, 50 μg/ml kanamycin and 25 μg/ml gentamicin) for 24 h with continuous shaking at 28 °C, collected by centrifugation and resuspended in infiltration buffer (10 mM MgCl_2_, 10 mM MES, 200 μM acetosyringone, pH5.7). For transient expression, agrobacteria carrying different constructs were infiltrated into leaves of 4-week-old *N. benthamiana* plants using 1 mL needleless syringes. Leaf samples were collected 2 days after agroinfiltration for analyzing the expression level of the target genes by qRT-PCR, level of protein accumulation by Western blot or for disease assays.

For disease assays, inoculation of *B. cinerea* was performed using spore suspension (1 × 10^5^ spores/mL) according to previously reported procedure [16]. Briefly, detached leaves were inoculated by dropping a 5 μL of spore suspension and then kept in sealed trays at 22 °C to facilitate disease development. Disease progress was estimated by measuring the lesion sizes and fungal growth by qRT-PCR analyzing the transcript of *B. cinerea* ActinA gene as an indicative of fungal growth [16, 61] using a pair of primers BcActin-F and BcActin-R (Additional file 1: Table S1).

For Western blot analysis of the ClMPK7 protein, leaf discs were ground in 200 μL lysis buffer (50 mM Tris–HCl, pH7.4, 150 mM NaCl, 1 mM EDTA, 1 mM DDT, 0.1 % Triton X-100, and 1× protease inhibitor cocktail, 1 mM PMSF), followed by addition of 100 μL loading buffer. After boiling for 5 min, the samples were centrifuged at 10,000× *g* for 10 min at 4 °C and 20 μL of the supernatant were separated on a 12 % SDS-PAGE gel, followed by transferring onto PVDF membrane by semi-dry transfer. Detection of GFP was performed using a polyclonal rabbit anti-GFP antibody (1:5000 dilution; GenScript, Nanjing, China) and a Horseradish peroxidase-conjugated anti-rabbit antibody (1:10,000 dilution; GenScript, Nanjing, China) according to the manufacturer’s instructions. Proteins in SDS-PAGE gel were detected by SuperSignal West Pico Chemiluminescent Substrate (Thermo Scientific, Rockford, IL, USA).

### Detection of H_2_O_2_ accumulation

Detection of H_2_O_2_ was performed by DAB staining [62]. Leaf samples were collected from *N. benthamiana* plants at 48 h after infiltration for transient expression and dipped into DAB solution (1 mg/mL, pH3.8). After incubation for 8 h in dark at room temperature, the DAB-treated leaves were transferred into acetic acid/glycerol/ethanol (1:1:1, vol/vol/vol) and boiled for 5 min, followed by several washes with the same solution. The DAB-stained leaves were photographed using a digital camera.

### qRT-PCR analysis of gene expression

Total RNA was extracted by Trizol regent (TaKaRa, Dalian, China) according to the manufacturer’s instructions. RNA was treated with RNase-free DNase and then reverse-transcribed into cDNA using the PrimeScript RT regent kit (TaKaRa, Dalian, China). The obtained cDNAs were used for gene expression analysis with real time quantitative PCR. Each qPCR reaction contained 12.5 μL SYBR Premix Ex Taq (TaKaRa, Dalian, China), 0.1 μg cDNA and 7.5 pmol of each gene-specific primer (Additional file 1: Table S1) in a final volume of 25 μL, and had three independent biological replicates. The qPCR was performed in a CFX96 real-time PCR detection system (Bio-Rad, Hercules, CA, USA). Relative gene expression level was calculated using 2^–△△CT^ method as described.

### Statistical analysis

All experiments were repeated independently three times and data obtained from three independent experiments were subjected to statistical analysis according to the Student’s *t*-test. The probability values of *p* ≤ 0.05 were considered as significant difference between the treatments and corresponding controls.

## Availability of supporting data

Sequence information on the watermelon and Arabidopsis MPKs and MKKs used in phylogenetic trees can be found in the LabArchives database under DOI of 10.6070/H4HQ3WXB (https://mynotebook.labarchives.com/share/Dayong%2520Li/MjYuMHwxMDIwMDkvMjAvVHJlZU5vZGUvNzg5MzI4ODZ8NjYuMA==).

## Additional file

Additional file 1: Table S1. Primers used in this study for different purposes. (DOC 136 kb)

## Abbreviations

ABA: Abscisic acid
*B. cinerea*: *Botrytis cinerea*
DAB: 3, 3-diaminobenzidine
dpi: Days after inoculation
*Fon*: *Fusarium oxypsorum* f. sp. *niveum*
HR: Hypersensitive response
MAPK: Mitogen-activated protein kinase
MKK: MAPK kinase
MEKK: MKK kinase
*N. benthamiana*: *Nicotiana benthamiana*
ORF: Open reading frame
qRT-PCR: Quantitative reverse transcription PCR
