# Supplementary material for: Characterization, expression patterns and functional analysis of the MAPK and MAPKK genes in watermelon (Citrullus lanatus)
Source: BMC Plant Biol. 2015 Dec 23;15:298. doi: 10.1186/s12870-015-0681-4 (PMC5477810; doi:10.1186/s12870-015-0681-4)
Supplement: Additional file 1: Table S1. — Primers used in this study for different purposes. (DOC 136 kb) [file 12870_2015_681_MOESM1_ESM.doc]

**Additional file: Table S1** Primers used in this study for different puropses

| Primers | | Sequence (5’-3’) | Size (bp) |
| --- | --- | --- | --- |
| ***Cloning of ORFs*** | | | |
| ClMKK6-F | | ATGAAGACCAAGACGCCATTGAAGC | 1065 |
| ClMKK6-R | | TTATCTGGGAAAACTTACCGGA |
| ClMKK2-1-F | | ATGAAGAAGGATAGCTCCATG | 1090 |
| ClMKK2-1-R | | TTAGAAGGTTGCTAATGGAGAGC |
| ClMKK2-2-F | | ATGAGGAAGGGAGGCTTCAGC | 1100 |
| ClMKK2-2-R | | TCCTGTTTTGATCGAAACCTCT |
| ClMKK5-F | | ATGAGACCGCTCCAGCCGCCT | 1110 |
| ClMKK5-R | | TCTGTCTGTCTCAGGAAAGGCT |
| ClMPK4-1-F | | ATGGCTACTAAAGAATCGAGT | 1050 |
| ClMPK4-1-R | | TCAAACACAAAGAGTTGTCCT |
| ClMPK4-2-F | | ATGGAGTCCAGTTCTTTTGATC | 1140 |
| ClMPK4-2-R | | TCAAAAAGCGGGATCGGGGTTGAACT |
| ClMPK3-F | | ATGGCTGATGTTGCTCAGAAC | 1113 |
| ClMPK3-R | | ACTCAATCCAGAATTTGCATGA |
| ClMPK6-1-F | | ATGGACGATGGAGCAGCTTCTCAGC | 1200 |
| ClMPK6-1-R | | TAGCTTATTATTGGTGATGATACT |
| ClMPK13-F | | ATGGAGAATGATTCTTCTTCCGCT | 1120 |
| ClMPK13-R | | TGAAACTAAATGTGATTTGGAT |
| ClMPK16-F | | ATGATGCAGTCGTCTATAGATGT | 1700 |
| ClMPK16-R | | TCAGTACCAGTTATTTCCTGGT |
| ClMPK19-3-1F | | TCTCAAGAGCGGAATTTGAGT | 900 |
| ClMPK19-5-1R | | GGTGTCACTGAAAGCAACTCT |
| ClMPK1-F | | ATGGCGACCCCTGTAGAGCCTC | 1161 |
| ClMPK1-R | | TCAAGAATACACTGCAGTACT |
| ClMPK7-F | | ATGGCTACGTTCGTGGAGCCAC | 1107 |
| ClMPK7-R | | TCAATAATATCCTGAAACAGCT |
| ClMPK9-2-F | | ATGATTGAAAAGGAATTTTTCACT | 1600 |
| ClMPK9-2-R | | GTCAAATGGCAAGTTCAGAGGCGT |
| ***qRT-PCR*** | | | |
| ClMKK2-2-RT-F | | TCAGCAACAACCTCAATCTCAAGC | 93 |
| ClMKK2-2-RT-R | | CGCTTTGAGAAACAATACGAACCC |
| ClMKK5-RT-F | | CAGCTTCCGCAGAGGGACAATA | 158 |
| ClMKK5-RT-R | | TGGAGTTGACTGGTGGAAGGAG |
| ClMKK6-RT-F | | AGACCAAGACGCCATTGAAGC | 81 |
| ClMKK6-RT-R | | CACTCGCAGTCAGGAAAGAACG |
| ClMKK2-1-RT-F | | GCTCCATGAATCCTAACTTAACGC | 98 |
| ClMKK2-1-RT-R | | CAAATCGCCATCGGTGAAAGTA |
| ClMKK3-RT-F | | ATGGCGGGTCTTGAGGAACTT | 83 |
| ClMKK3-RT-R | | TCCACAGGGGAGTCCGTAGAAA |
| ClMKK9-RT-F | | GATATTTGGAGTTTGGGGCT | 166 |
| ClMKK9-RT-R | | CAACAAAGCTTCGAAACTCC |
| ClMPK9-3-RT-F | | GGCAGTCCAATTTTAAGGAAGC | 182 |
| ClMPK9-3-RT-R | | TGAACAGAACCGTTTCCAGCA |
| ClMPK9-2-RT-F | | GCAAGCAAGCCTTTGTTTCCTGGG | 194 |
| ClMPK9-2-RT-R | | AGAGCTAGTGGATCTGCATTCGGGA |
| ClMPK20-2-RT-F | | ACATGACACGTGCCGGAGTG | 142 |
| ClMPK20-2-RT-R | | ACCTTTCTGTGAGCAACCGA |
| ClMPK19-RT-F | | CATCCTCCACCTAAAGTGCCTAC | 157 |
| ClMPK19-RT-R | | CCCTACTGTTGGTTATGCCTATCTC |
| ClMPK20-1-RT-F | | GCAGGTAAGTGCTCAATATGATGC | 99 |
| ClMPK20-1-RT-R | | CACATTCTTGTCATGCCATACTCG |
| ClMPK4-2-RT-F | | ATGGAGTCCAGTTCTTTTGATCAGA | 70 |
| ClMPK4-2-RT-R | | CATTGTATTGAATGTATCGACCACC |
| ClMPK3-RT-F | | GGACTCATCGCTGAGAAGAACTG | 73 |
| ClMPK3-RT-R | | GGTCAAAGGTTCCAGCAGAGTG |
| ClMPK13-RT-F | | ATGGAGAATGATTCTTCTTCCGC | 90 |
| ClMPK13-RT-R | | GAAGTTGCCGAGAACATTGTAGAG |
| ClMPK16-RT-F | | CCTTCCATTCCACCACAGTACG | 157 |
| ClMPK16-RT-R | | CTTGGGCAGCAGCAACTTTTC |
| ClMPK4-1-RT-F | | AGAATCGAGTTCTACCACTCCCACT | 122 |
| ClMPK4-1-RT-R | | GAGGGACATACTTAGCCGAAACC |
| ClMPK7-RT-F | | TTCTCGAGAGGAATACGCCTT | 163 |
| ClMPK7-RT-R | | GGTTGAATCTCGGGTCGTAT |
| ClMPK9-4-RT-F | | GGGGTAACACCAAAGGAGAAGTC | 83 |
| ClMPK9-4-RT-R | | TTCTGAGACAAGCCATCCACTG |
| ClMPK9-1-RT-F | | AACAAGCCAAATTACAGTGCCC | 168 |
| ClMPK9-1-RT-R | | TCAAGTATGCAAGGCCGACAC |
| ClMPK6-1-RT-F | | ATGGACGATGGAGCAGCTTCTC | 103 |
| ClMPK6-1-RT-R | | GCTGATGCTGCTGGTGCG |
| ClMPK1-RT-F | | GCTTCATTACCACCCCGAGGAT | 73 |
| ClMPK1-RT-R | | ACTTGTAGTATGTTCGGGGTGGA |
| ClGAPDH-F | | ATGGGCAAAGTTAAGATCGGCATCA | 91 |
| ClGAPDH-R | | CCAATTCGATATCATCACTCTGC |
| ClPR5-F | | CCTTCTCTTCACCTTCTCTGCT | 129 |
| ClPR5-R | | CCCTGTGGAGTCTCTTTGAAAC |
| ClChitinase-F | | ATGACGTTATTGTCGGGCGATGGC | 109 |
| ClChitinase-R | | CCGCACTCGAGTCCACCGTTAAT |
| BcActin-RT-F | | CGTCACTACCTTCAACTCCATC | 107 |
| BcActin-RT-R | | CGGAGATACCTGGGTACATAGT |
| NbActin-RT-F | | ACCAGATTAATGAGCCCAAGAG | 97 |
| NbActin-RT-R | | CCAACAGGGACAGTACCAATAC |
| NbPR1-RT-F | | CCGTTGAGATGTGGGTCAAT | 100 |
| NbPR1-RT-R | | CGCCAAACCACCTGAGTATAG |
| NbPR2-RT-F | | CAACCCGCCCAAAGATAGTA | 98 |
| NbPR2-RT-R | | TGGCTAAGAGTGGAAGGTTATG |
| NbPR5-RT-F | | GCTCGATTACGTCTTGTCTCTC | 104 |
| NbPR5-RT-R | | CTCTAGCATGGTGGATTGACTT |
| ***Yeast two-hybrids*** | | | |
| ClMPK9-2-pGADT7-F | | CCGGAATTC ATGATTGAAAAGGAATTTTTC | 1422 |
| ClMPK9-2-pGADT7-R | | CGCGGATCC TTAATCCACCCATTTAGAACA |
| ClMPK4-2-pGADT7-F | | CCGGAATTC ATGGAGTCCAGTTCTTTTGAT | 1140 |
| ClMPK4-2-pGADT7-R | | CGCGGATCCTCAAAAAGCGGGATCGGGGTT |
| ClMPK13-pGADT7-F | | CCGGAATTC ATGGAGAATGATTCTTCTTCC | 1113 |
| ClMPK13-pGADT7-R | | CGCGGATCC CTAAATGTGATTTGGATTGAA |
| ClMPK16-pGADT7-F | | TCCCCCGGG ATGATGCAGTCGTCTATAGAT | 1686 |
| ClMPK16-pGADT7-R | | CGCGGATCC TCAGTACCAGTTATTTCCTGG |
| ClMPK4-1-pGADT7-F | | CCGGAATTC ATGGCTACTAAAGAATCGAGT | 1152 |
| ClMPK4-1-pGADT7-R | | CGCGGATCC TCAAACACAAAGAGTTGTCCT |
| ClMPK7-pGADT7-F | | CCGGAATTC ATGGCTACGTTCGTGGAGCCA | 1107 |
| ClMPK7-pGADT7-R | | CGCGGATCC TCAATAATATCCTGAAACAGC |
| ClMPK6-1-pGADT7-F | | CCGGAATTC ATGGACGATGGAGCAGCTTCT | 1218 |
| ClMPK6-1-pGADT7-R | | CGCGGATCC TCACTCACTGACGTTTTCATG |
| ClMPK1-pGADT7-F | | CCGGAATTC ATGGCGACCCCTGTAGAGCCT | 1161 |
| ClMPK1-pGADT7-R | | CGCGGATCC TCAAGAATACACTGCAGTACT |
| ClMKK2-2-pGBKT7-F | | CGCCATATG ATGAGGAAGGGAGGCTTCAGC | 1023 |
| ClMKK2-2-pGBKT7-R | | CGCGGATCC TTAGAAAGTTGCAAGTGGAGA |
| ClMKK5-pGBKT7-F | | CGCCATATG ATGAGACCGCTCCAGCCGCCT | 1110 |
| ClMKK5-pGBKT7-R | | CGCGGATCC TCAGGAAAGGCTGGAAGGATG |
| ClMKK6-pGBKT7-F | | CCGGAATTC ATGAAGACCAAGACGCCATTG | 1065 |
| ClMKK6-pGBKT7-R | | GCGTCGAC TTATCTGGGAAAACTTACCGG |
| ClMKK2-1-pGBKT7-F | | CCGGAATTC ATGAAGAAGGATAGCTCCATG | 1086 |
| ClMKK2-1-pGBKT7-R | | GCGTCGAC TTAGAAGGTTGCTAATGGAGA |
| ***Transient expression*** | | | |
| ClMPK4-2-GFP-F | CGCGGATCCATGGAGTCCAGTTCTTTTGA | | 1140 |
| ClMPK4-2-GFP-R | TGCTCTAGATCAAAAAGCGGGATCGGGGT | |
| ClMPK7-GFP-F | CGCGGATCCATGGCTACGTTCGTGGAGCC | | 1107 |
| ClMPK7-GFP-R | TGCTCTAGATCAATAATATCCTGAAACAG | |
| ClMPK3-GFP-F | CGCGGATCCATGGTAAAGTGCTACCCTAC | | 1899 |
| ClMPK3-GFP-R | TGCTCTAGA TCATGCAAATTCTGGATTGA | |
| ClMPK19-GFP-F | CGCGGATCC ATGCAAACTGGGGAGAAAGT | | 1413 |
| ClMPK19-GFP-R | TCCCCCGGG CTACAACAATTCCATTTCAA | |
| ClMKK5-GFP-F | CGCGGATCC ATGAGACCGCTCCAGCCGCC | | 1110 |
| ClMKK5-GFP-R | TGCTCTAGA TCAGGAAAGGCTGGAAGGAT | |
| ClMPK6-1-GFP-F | CCCGGATCC ATGGACGATGGAGCAGCTTC | | 1218 |
| ClMPK6-1-GFP-R | GGGCCCGGG TTATTGGTGATGATACTCGGGG | |
| ClMKK2-1-GFP-F | CCCGGATCCATGAAGAAGGATAGCTCCATGAATC | | 1086 |
| ClMKK2-1-GFP-R | CCCTCTAGATTAGAAGGTTGCTAATGGAGAGC | |
| ClMPK1-GFP-F | CGCGGATCC ATGGCGACCCCTGTAGAGCC | | 1161 |
| ClMPK1-GFP-R | TGCTCTAGA TCAAGAATACACTGCAGTAC | |
| ClMPK9-2-GFP-F | CGCGGATCC ATGATTGAAAAGGAATTTTT | | 1422 |
| ClMPK9-2-GFP-R | TCCCCCGGG TTAATCCACCCATTTAGAAC | |
| ClMPK13-GFP-F | CGCGGATCC ATGGAGAATGATTCTTCTTC | | 1113 |
| ClMPK13-GFP-R | TCCCCCGGG CTAAATGTGATTTGGATTGA | |
| ClMPK4-1-GFP-F | CGCGGATCC ATGGCTACTAAAGAATCGAG | | 1017 |
| ClMPK4-1-GFP-R | TCCCCCGGG TCAAACACAAAGAGTTGTCC | |
| ClMKK2-2-GFP-F | CGCGGATCC ATGAGGAAGGGAGGCTTCAG | | 1023 |
| ClMKK2-2-GFP-R | TGCTCTAGA TTAGAAAGTTGCAAGTGGAG | |
| ClMKK6-GFP-F | TGCTCTAGA ATGAAGACCAAGACGCCATT | | 1065 |
| ClMKK6-GFP-R | TCCCCCGGG TTATCTGGGAAAACTTACCG | |
